# Supplementary figures and images for: Scale-Adjusted Metrics for Predicting the Evolution of Urban Indicators and Quantifying the Performance of Cities
Source: PLoS One. 2015 Sep 10;10(9):e0134862. doi: 10.1371/journal.pone.0134862 (PMC4565645; doi:10.1371/journal.pone.0134862)

$\log_{10}(Y_i)$

Child labor

Elderly pop.

Female pop.

Homicides

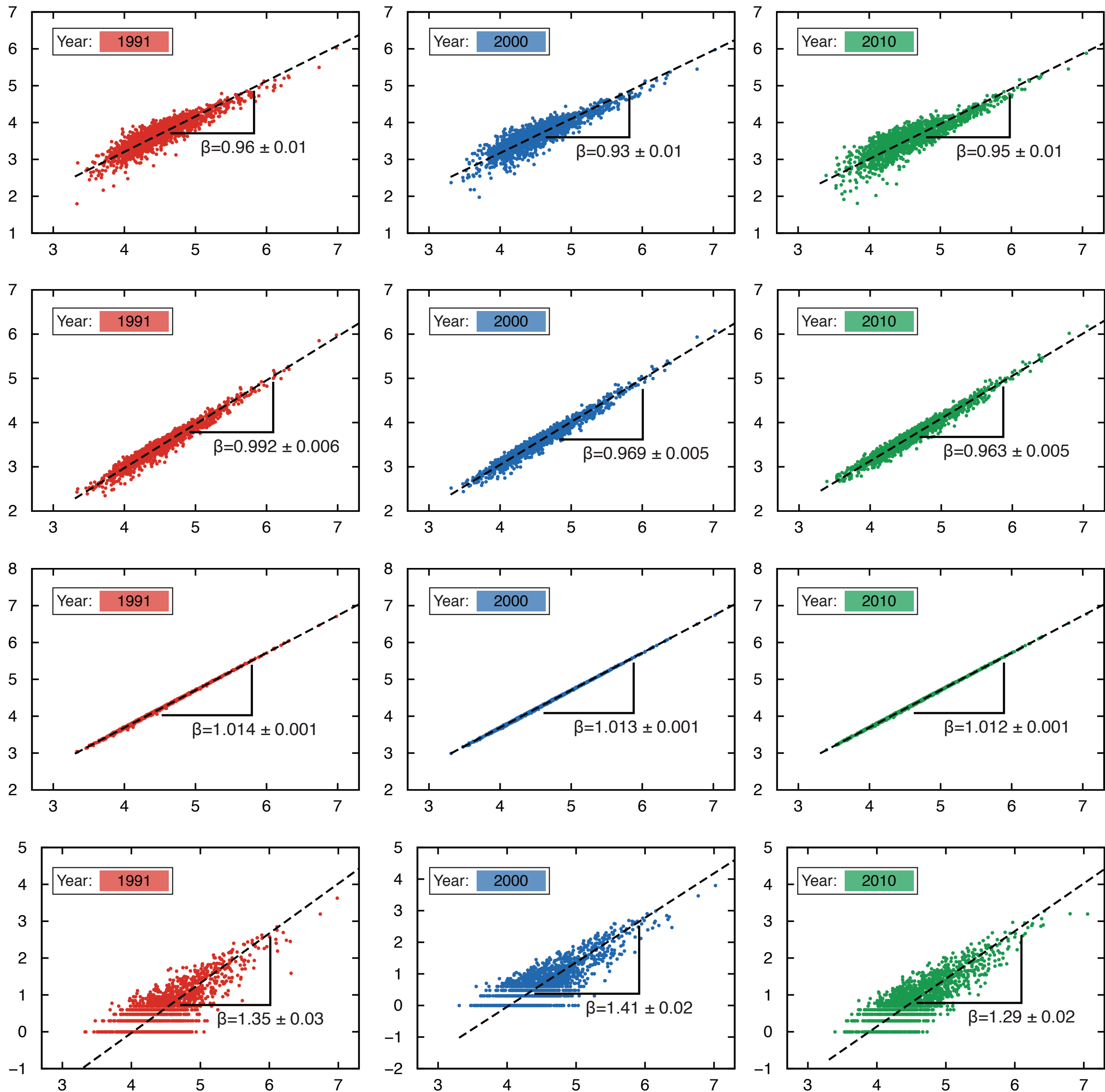

Population,  $\log_{10}(N)$

Supplement: S1 Fig — The scatter plots show the allometric relationships between the urban indicators (from top to bottom: child labor, elderly population, female population and homicides) and population size for the years t = 1991 (red dots), 2000 (blue dots) and 2010 (green dots) in log-log scale. The allometric exponents β i (see Methods Section for details on the calculation of β i) are shown in the figures. See S2 Fig for the other indicators. (PDF) [file pone.0134862.s002.pdf]

$\log_{10}(Y_i)$

Illiteracy

Family income

Male pop.

Unemployment

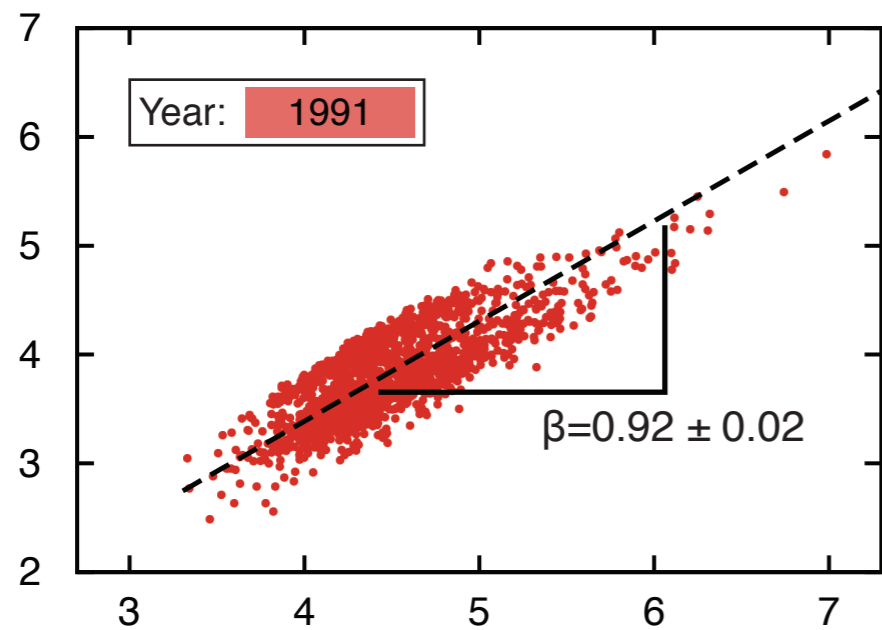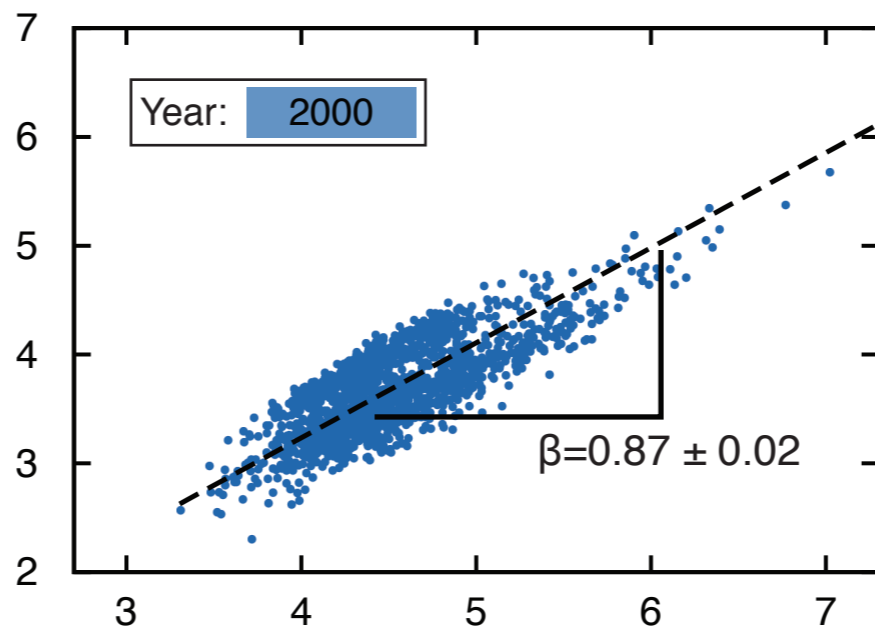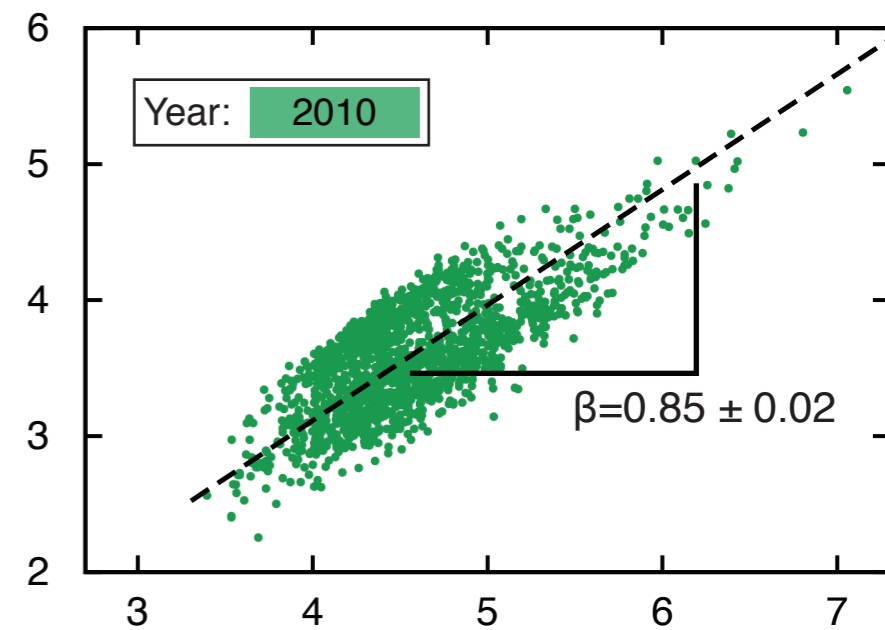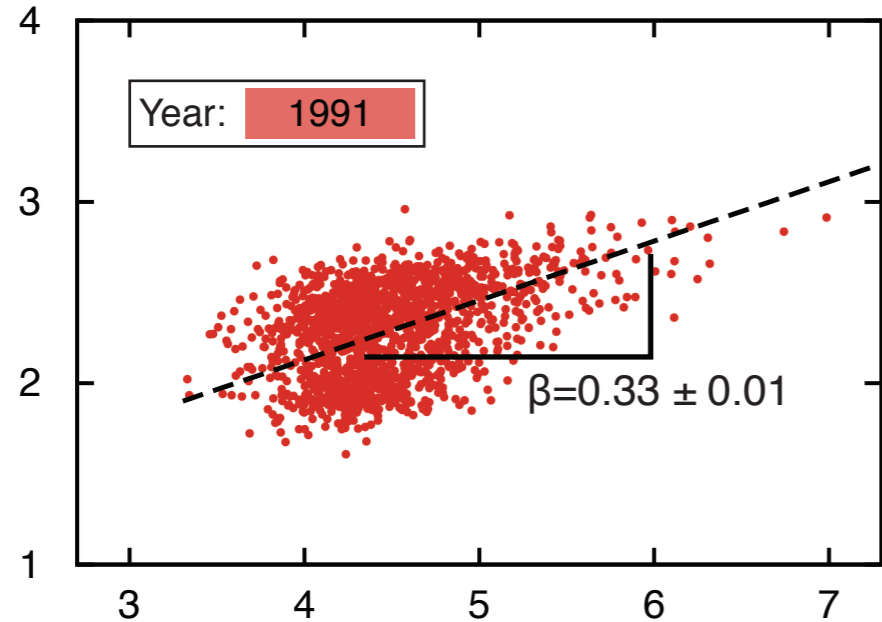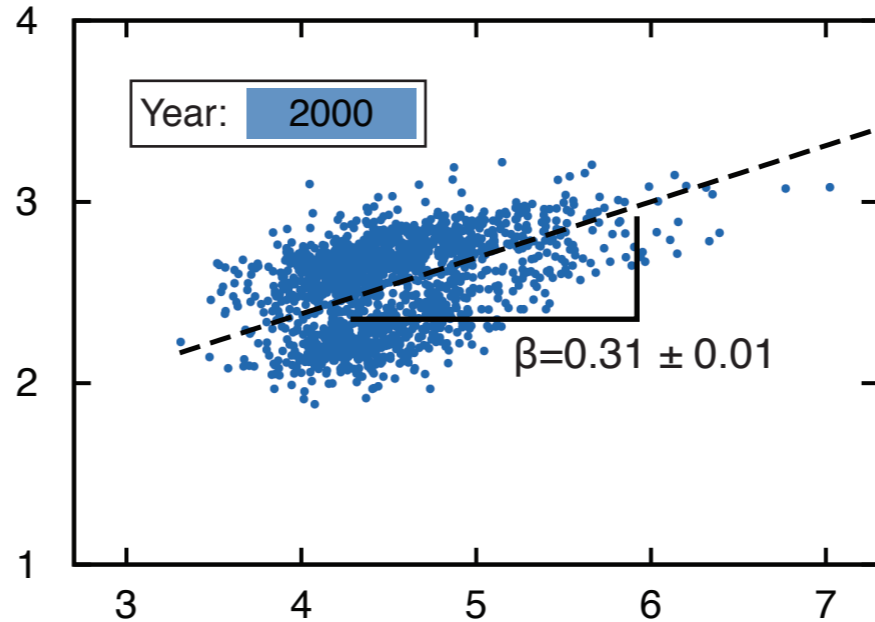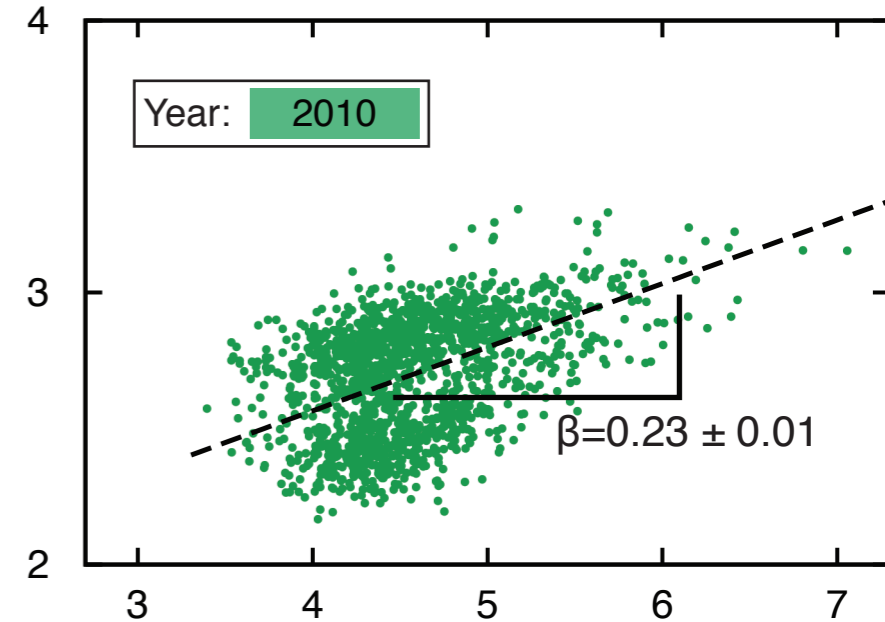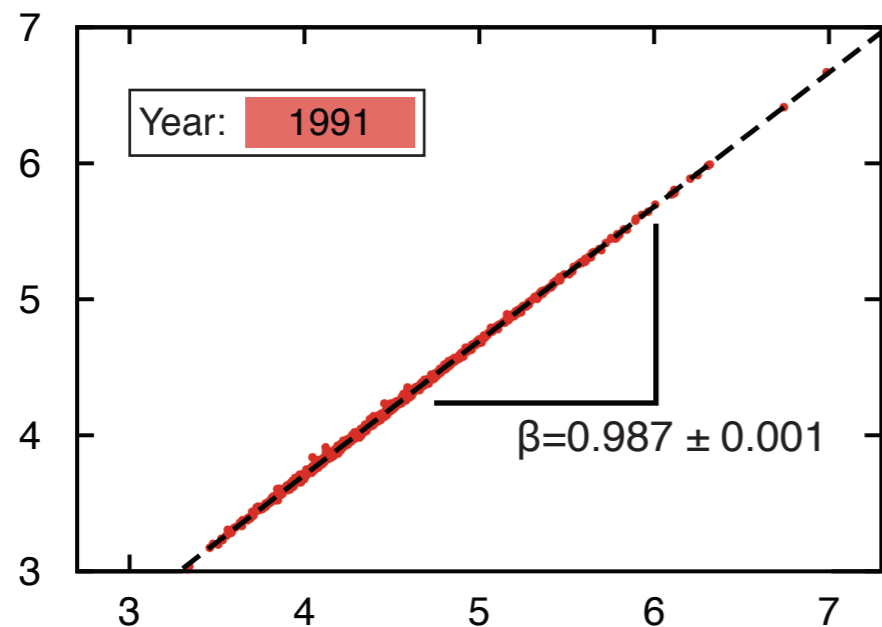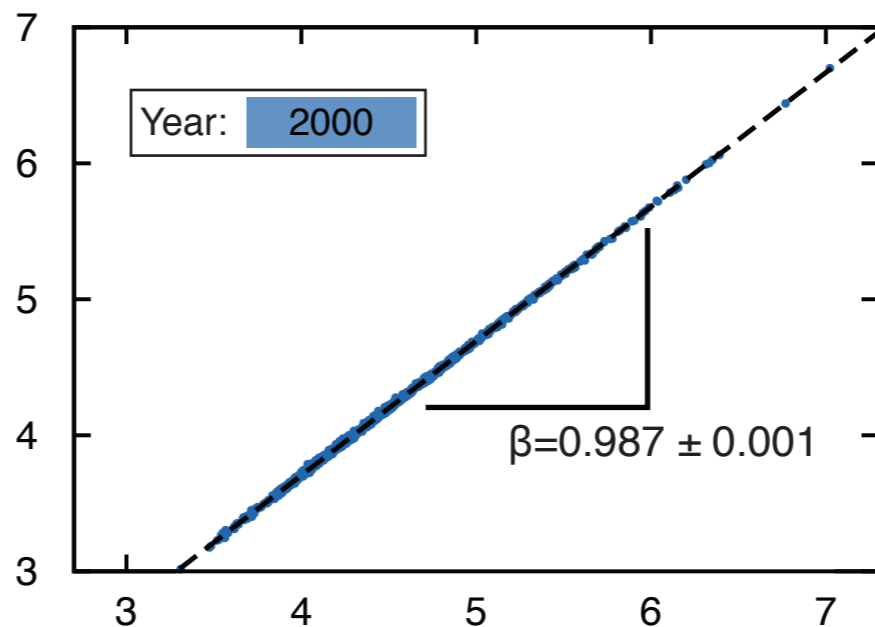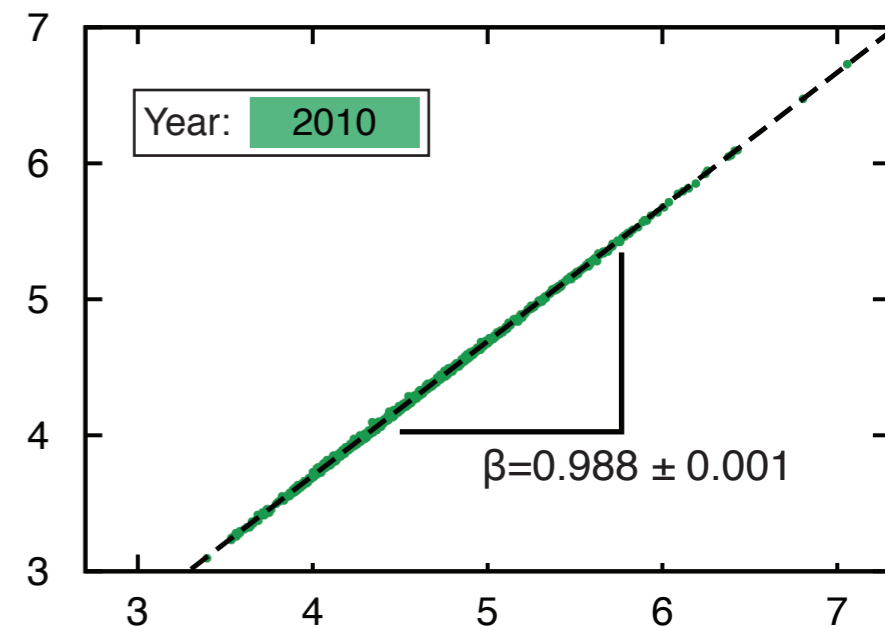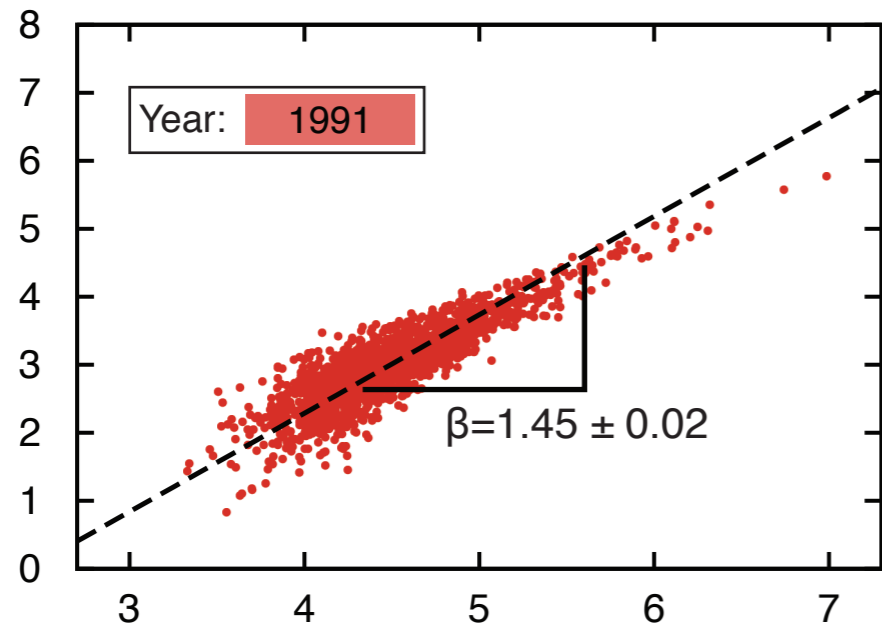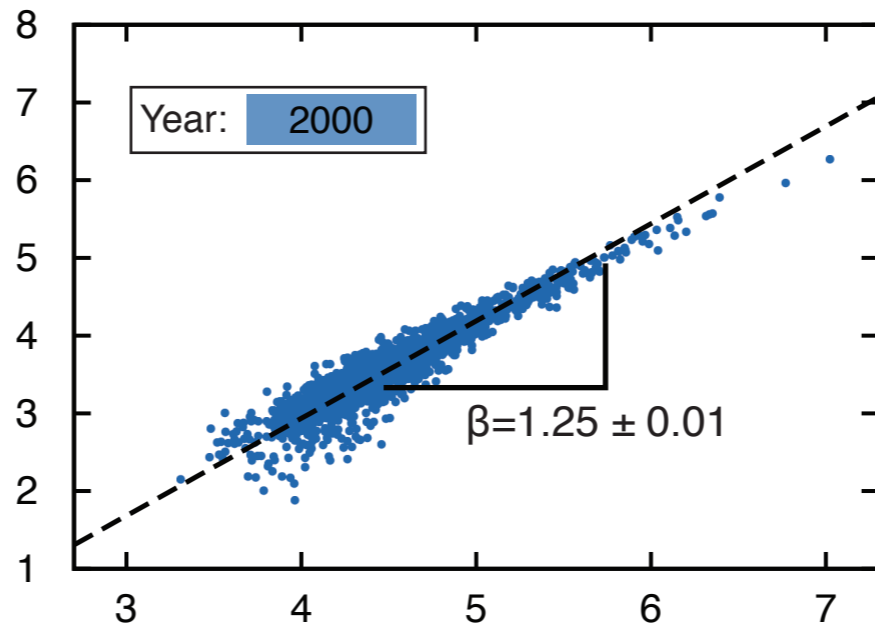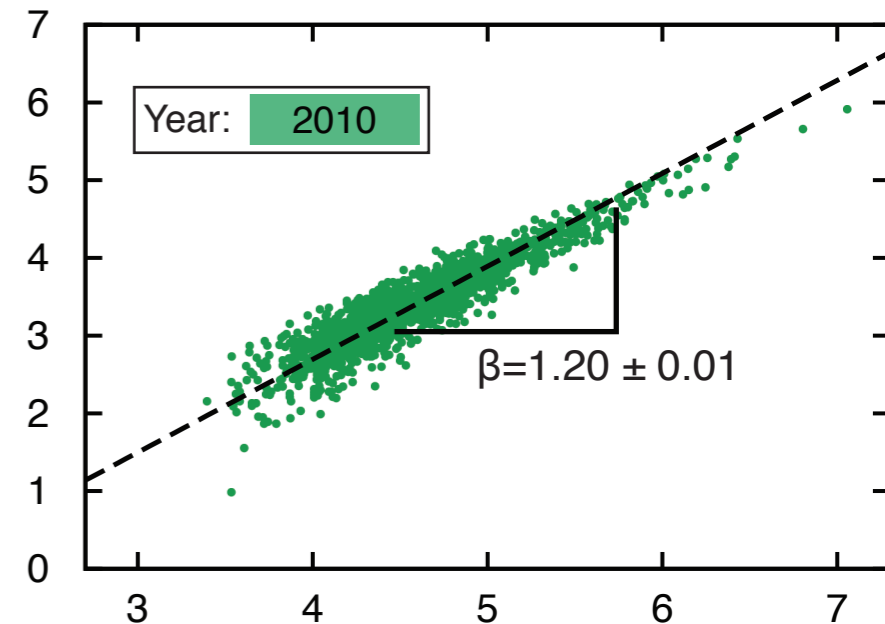

Population,  $\log_{10}(N)$

Supplement: S2 Fig — The same as S1 Fig for the indicators illiteracy, family income, male population and unemployment. (PDF) [file pone.0134862.s003.pdf]

2000

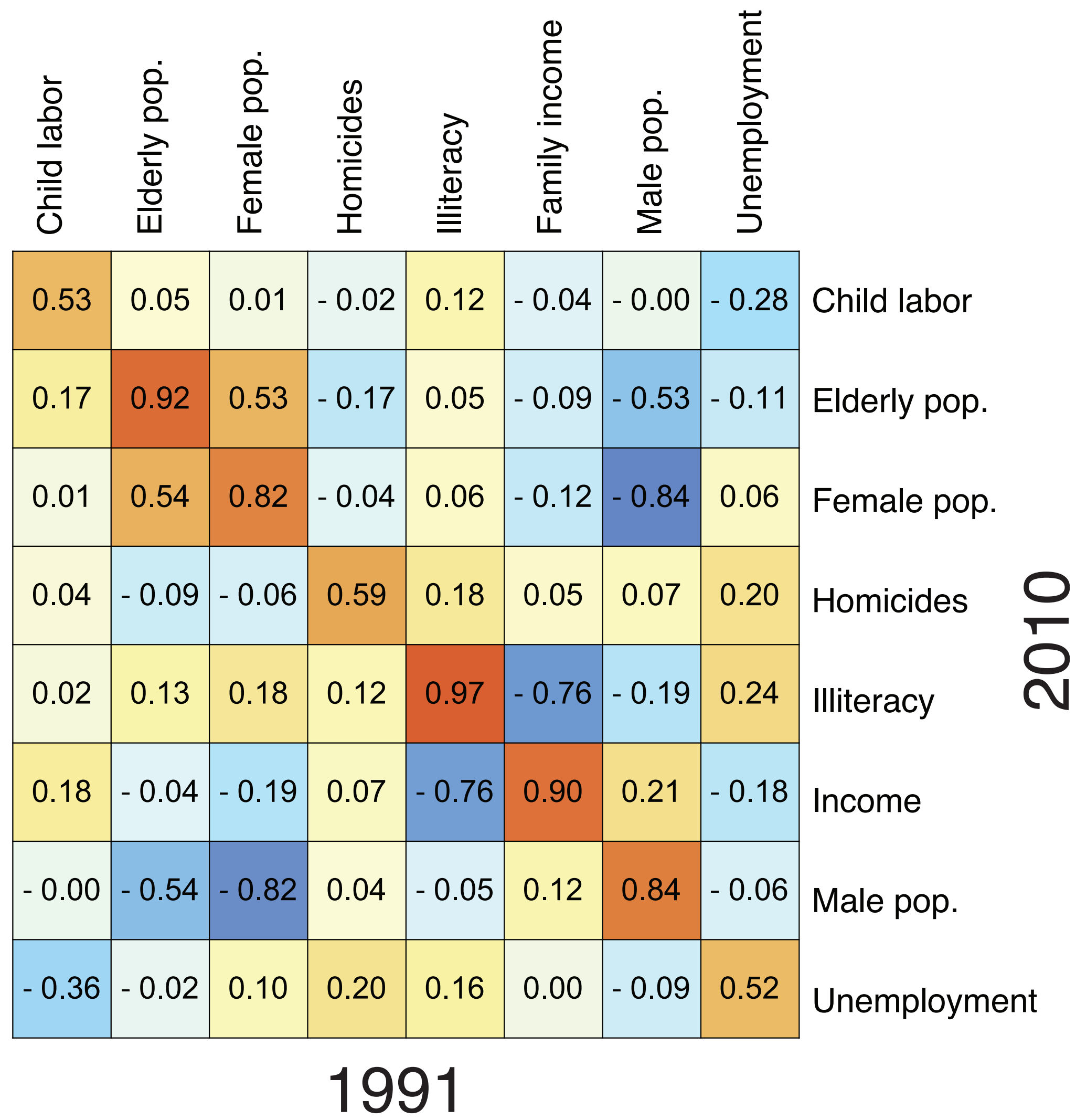

2010

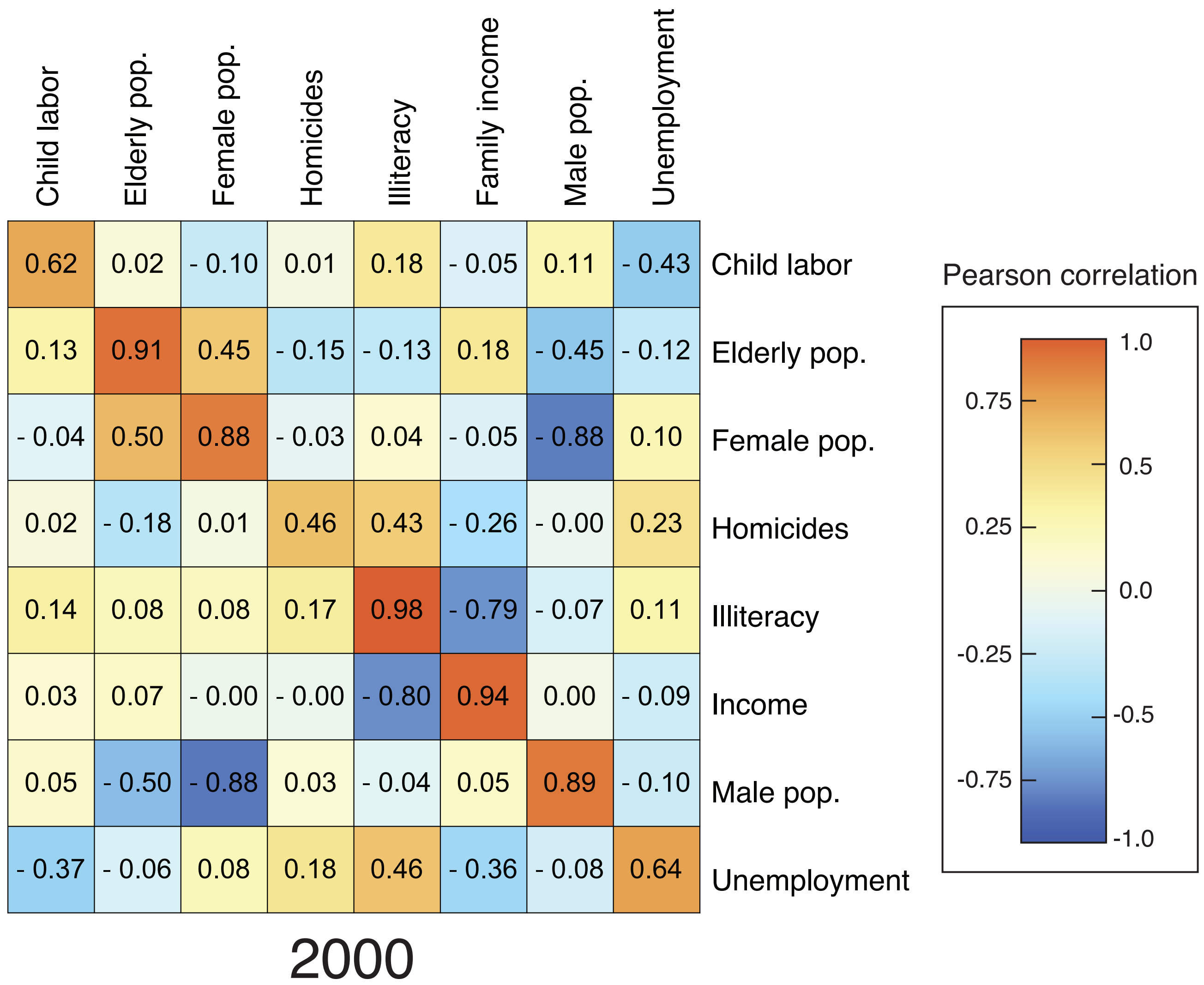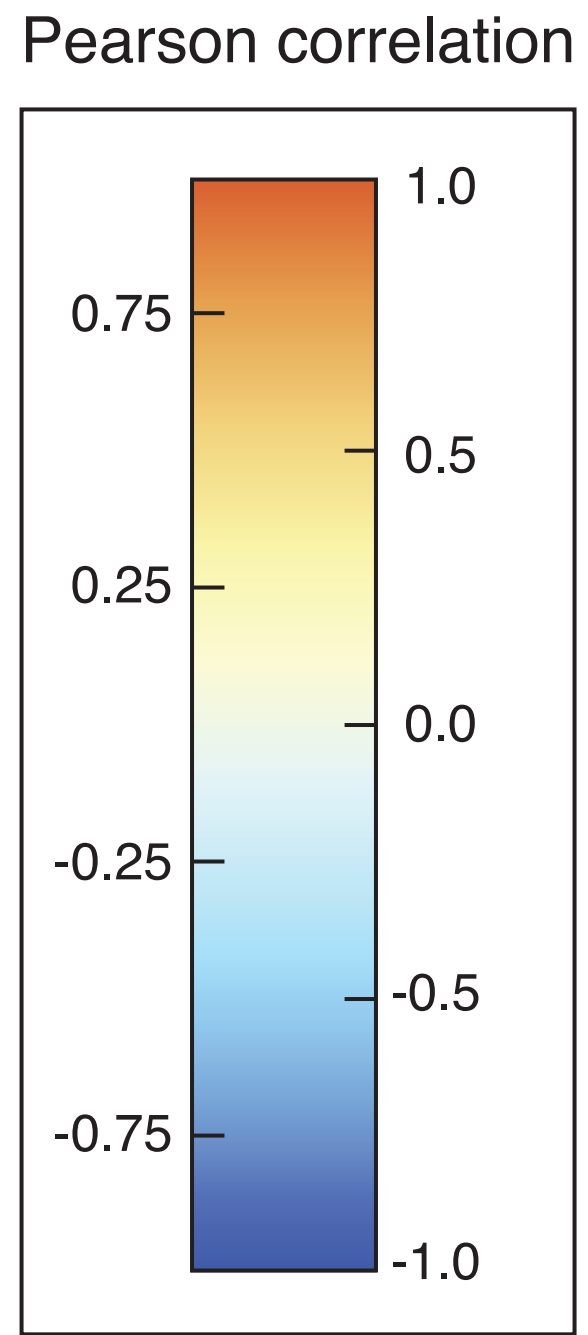

Supplement: S4 Fig — The matrix plot on left shows the values of the Pearson correlation coefficient between the scale-adjusted metric D Yi(t) for a given indicator (one indicator per row) in the year t = 2000 and all the other indicators in the year t = 1991 (one indicator per column). The right panel does the same for the years t = 2010 and t = 2000. The value inside each cell is the Pearson correlation and each one is also colored according to this value. We note that all indicators are strongly correlated with their own past values; furthermore, all indicators also display relevant correlations with at least one other indicator. (PDF) [file pone.0134862.s005.pdf]

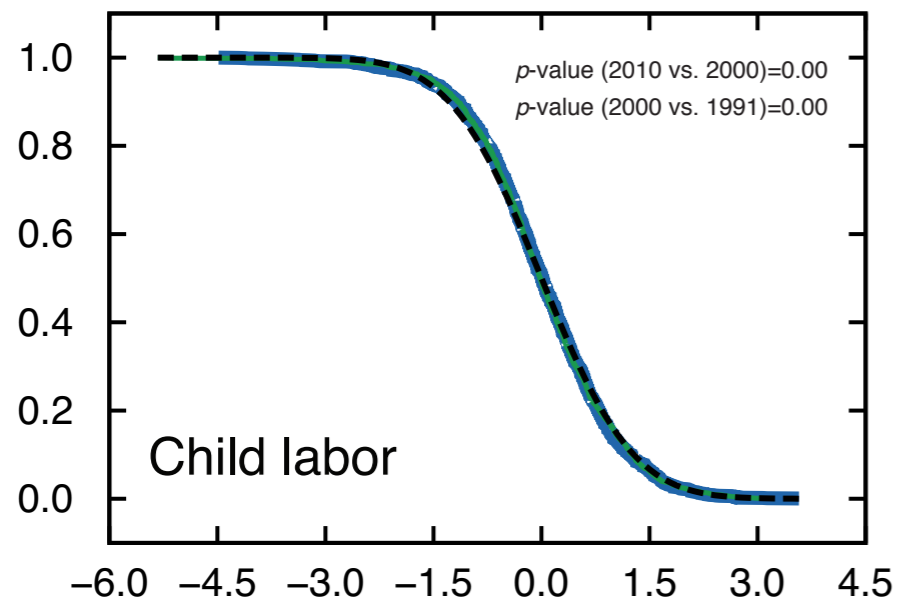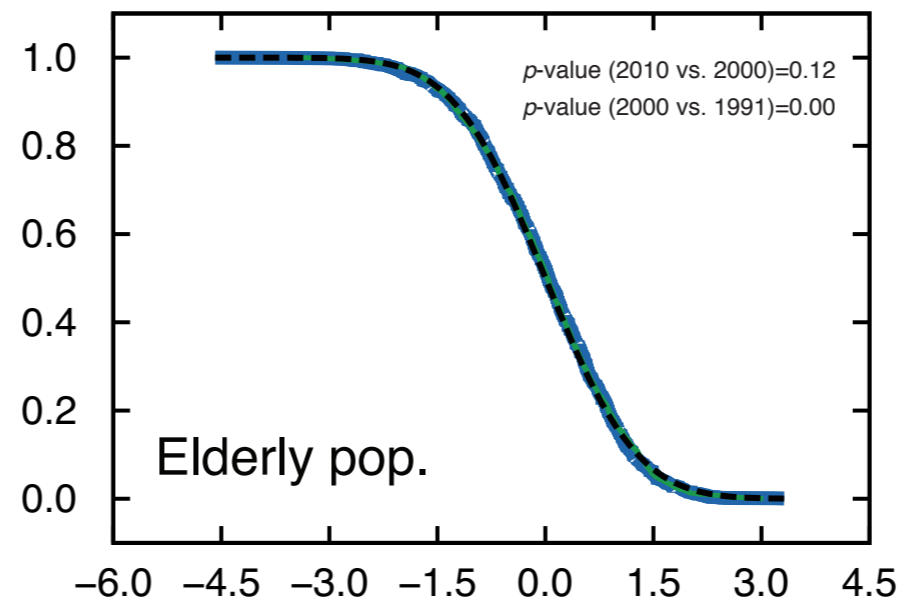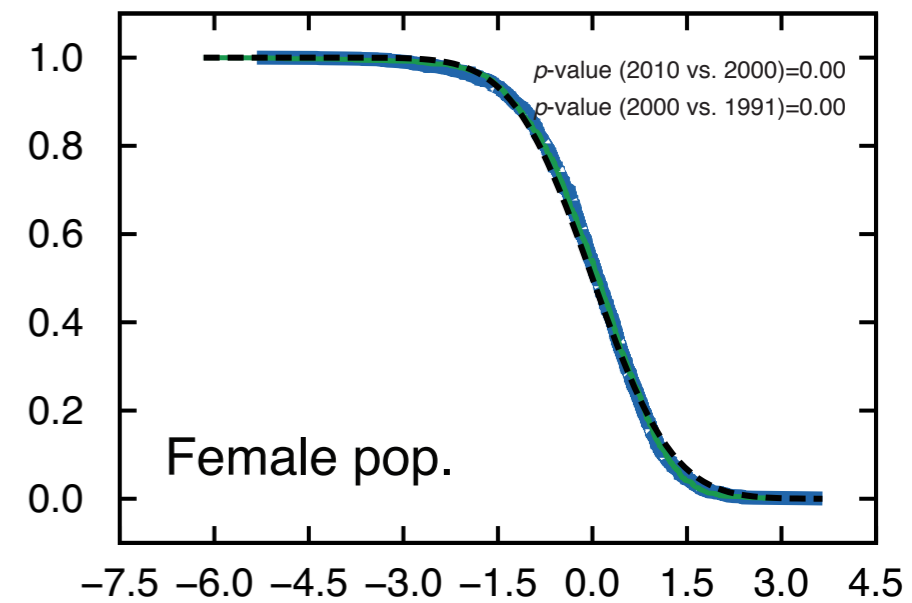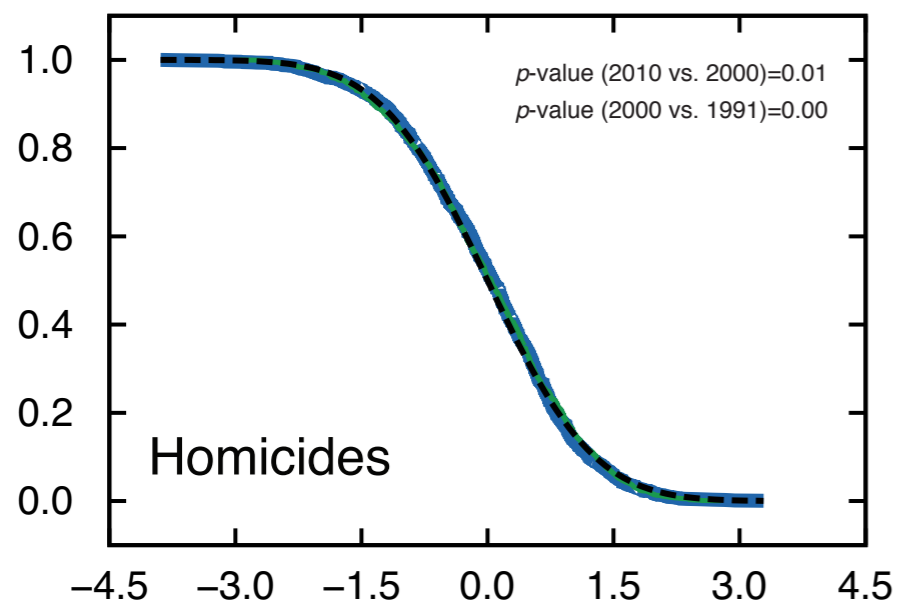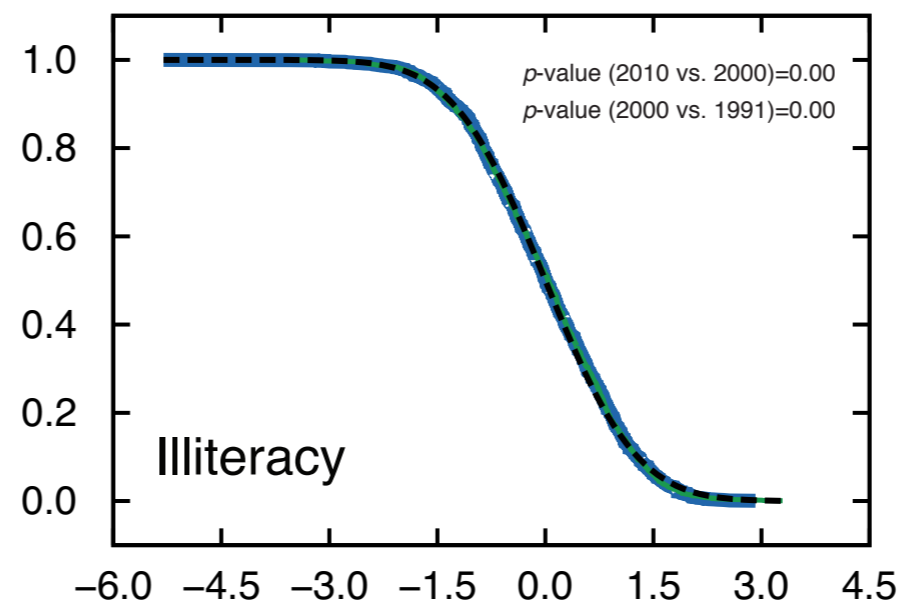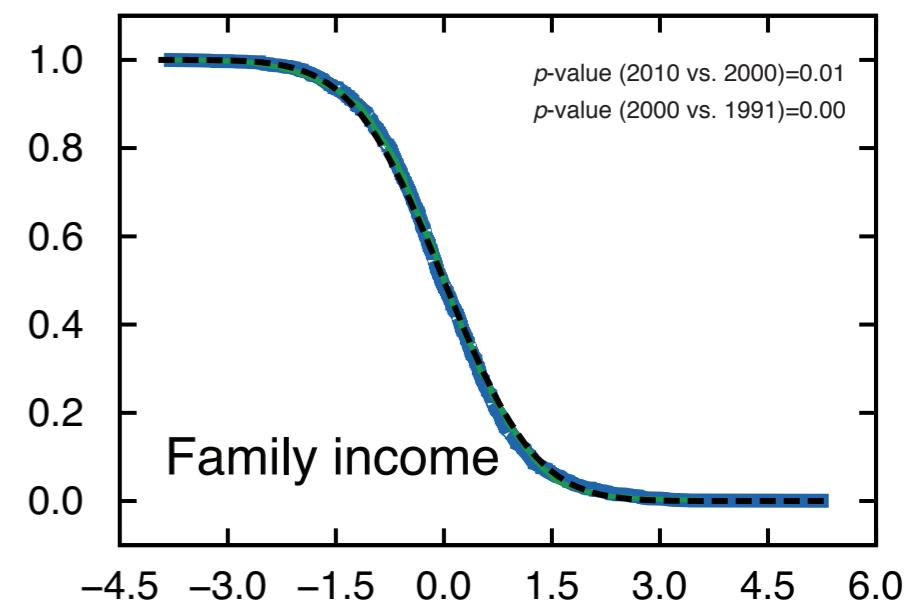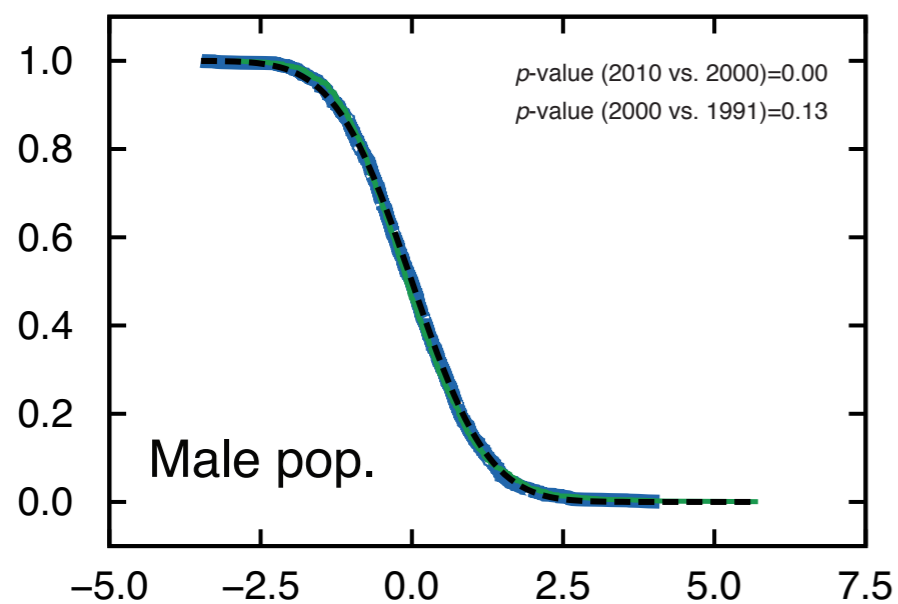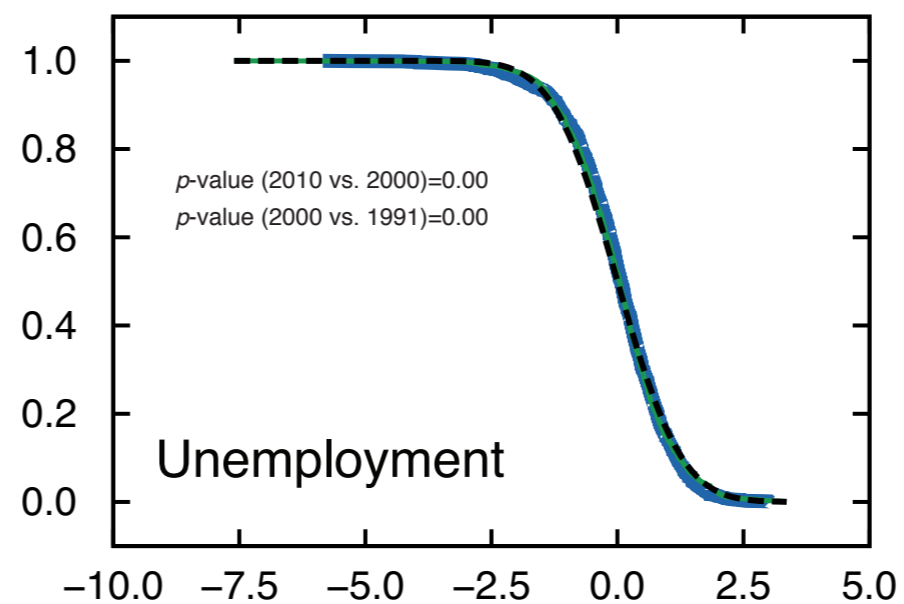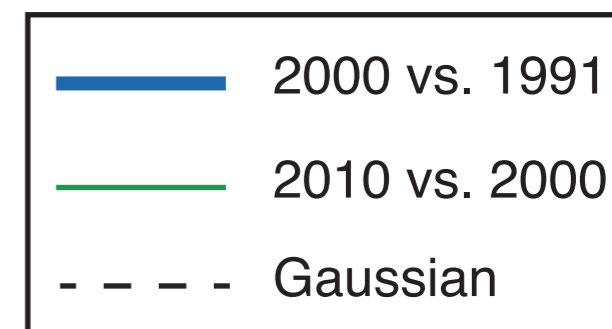

Supplement: S5 Fig — The plots show the cumulative distributions of the normalized residuals ξ of the linear regressions between D Yi(t + Δt) and D Yi(t) (Fig 4 and S3 Fig) for the years 2000-1991 (blue lines) and 2010-2000 (green lines) in comparison with the standard Gaussian (dashed lines). We also show the p-values of the Cramér von Mises method for testing the null hypotheses that the residuals ξ are normally distributed. We observe that the normality of the data is rejected in most cases (probably due the small heteroskedasticity present in these relationships—see S6 Fig). However, no huge differences are observed between the Gaussian cumulative curve and the empirical cumulative distributions, suggesting that ξ can be approximately described as a standard Gaussian noise. (PDF) [file pone.0134862.s006.pdf]

CDF

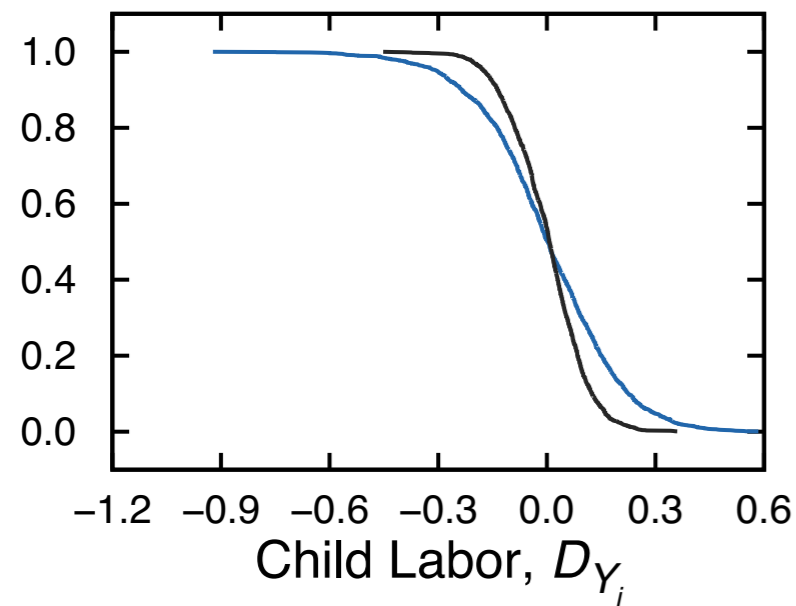

CDF

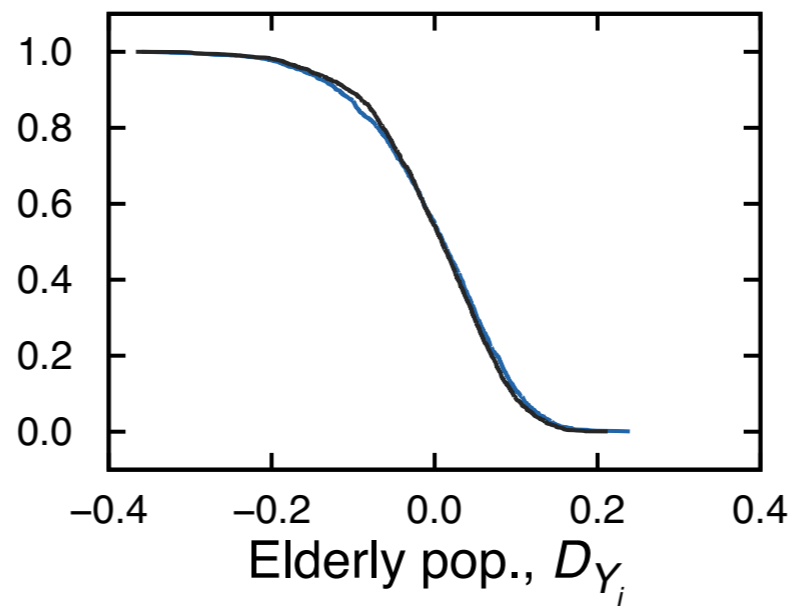

CDF

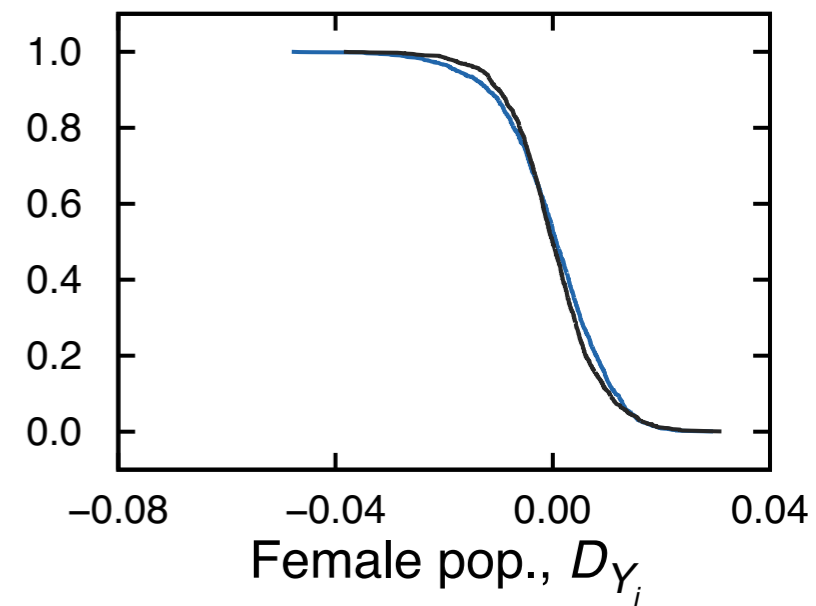

CDF

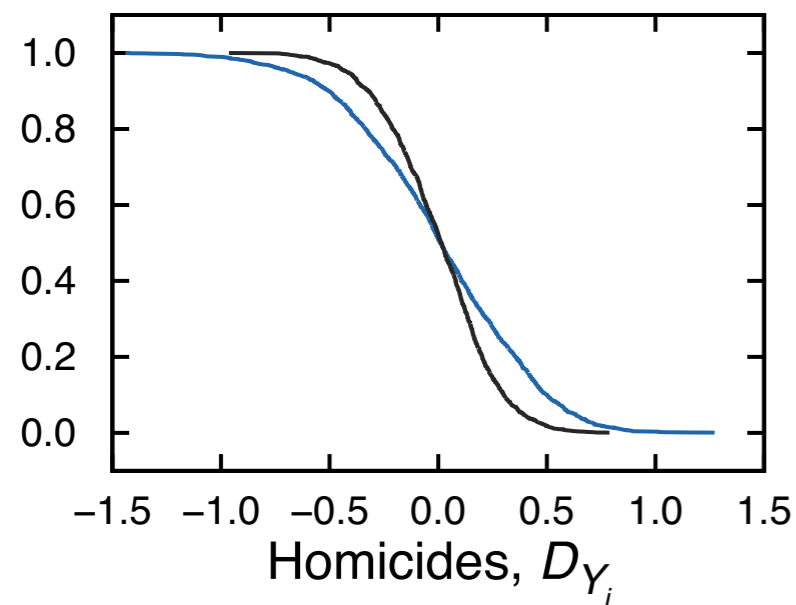

CDF

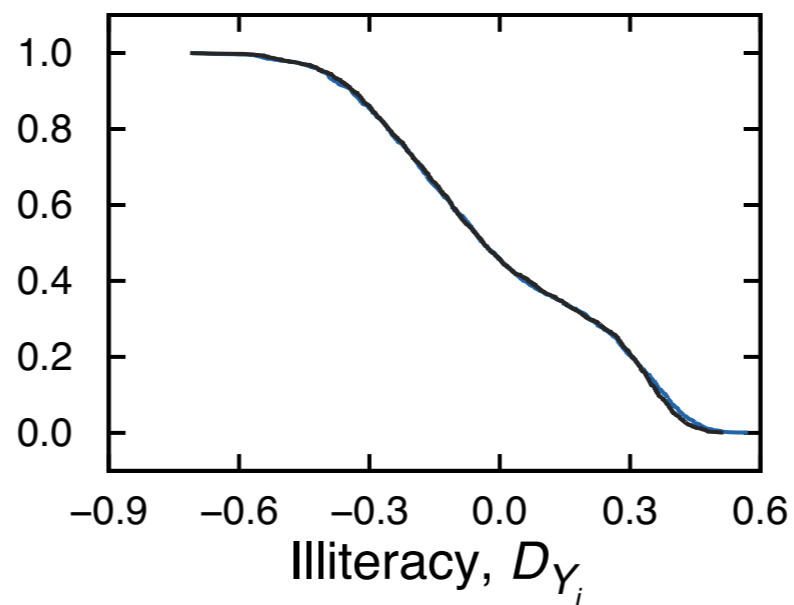

CDF

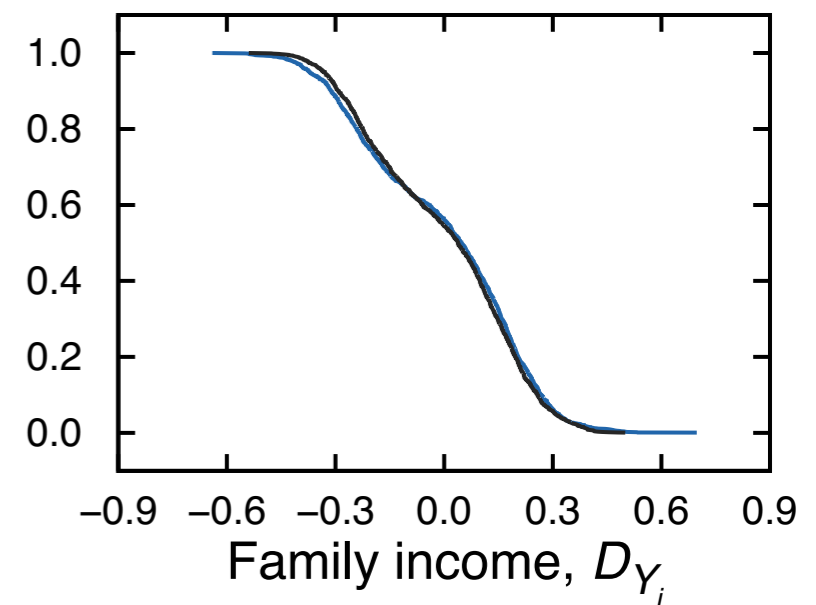

CDF

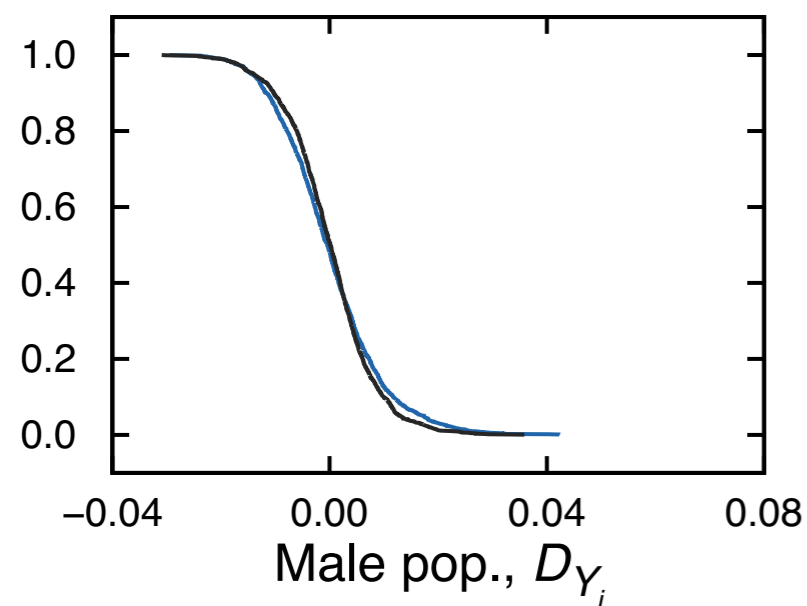

CDF

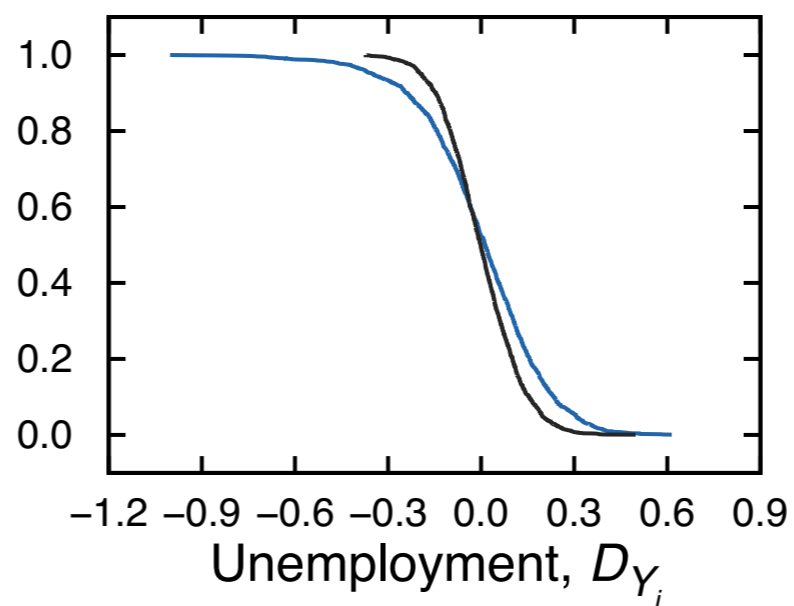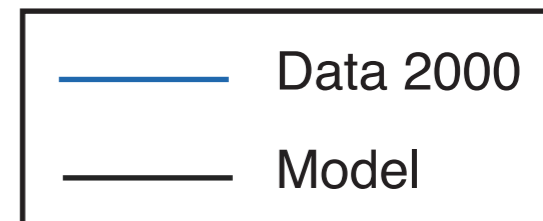

Supplement: S8 Fig — We have obtained the values of D Yi(t) in the year of 2000 using the linear model of Eq 6 and by employing data from the year of 1991. We thus calculated the cumulative distributions functions (CDF) of D Yi(t) for the predicted values (black lines) and the empirical ones (blue lines). We observe that the agreement is very good for the population indicators, illiteracy and family income; for the other indicators we observe that the model fails in reproducing the tails of the distributions. (PDF) [file pone.0134862.s009.pdf]

CDF

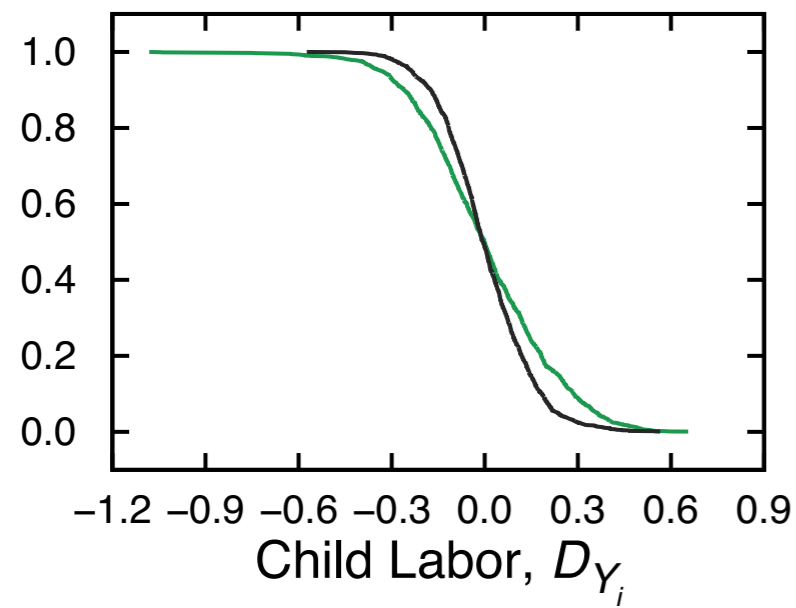

CDF

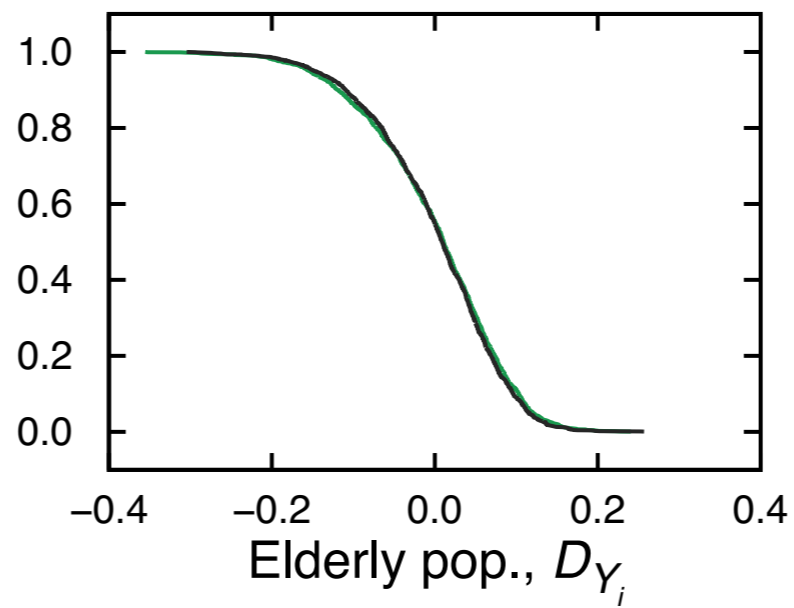

CDF

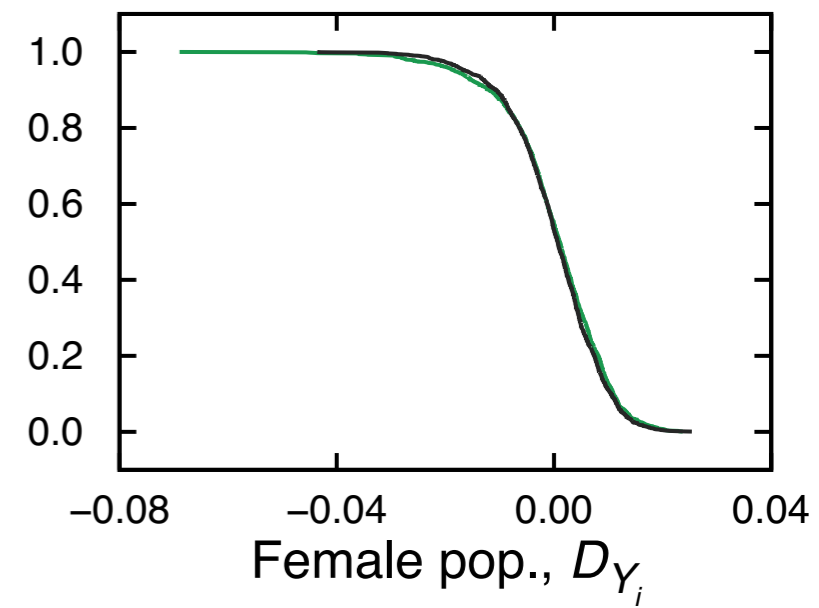

CDF

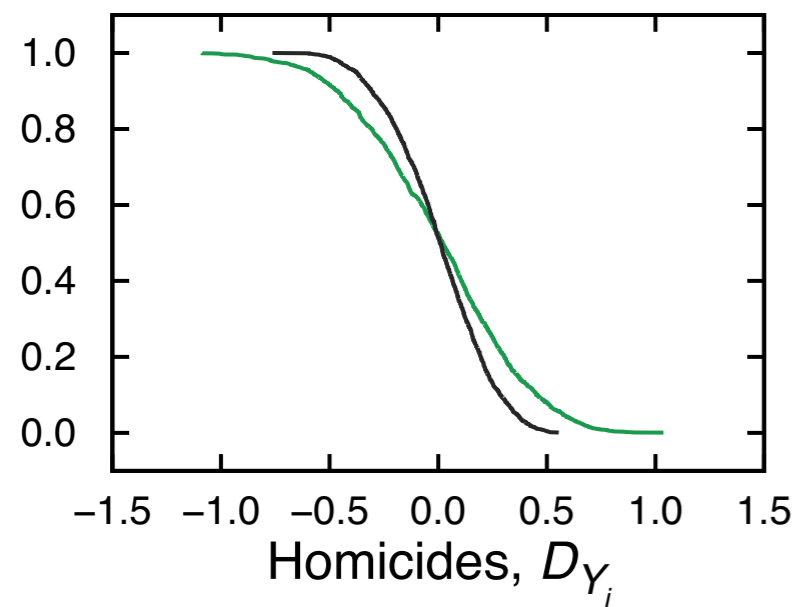

CDF

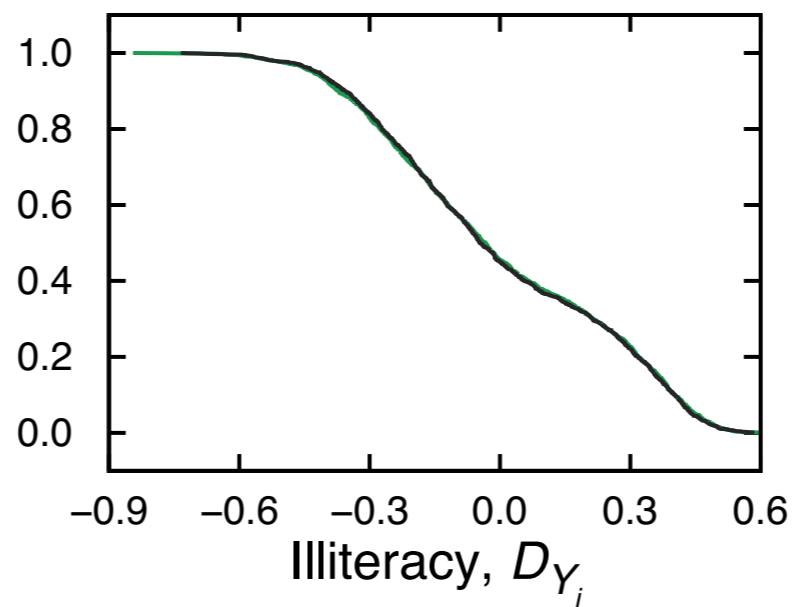

CDF

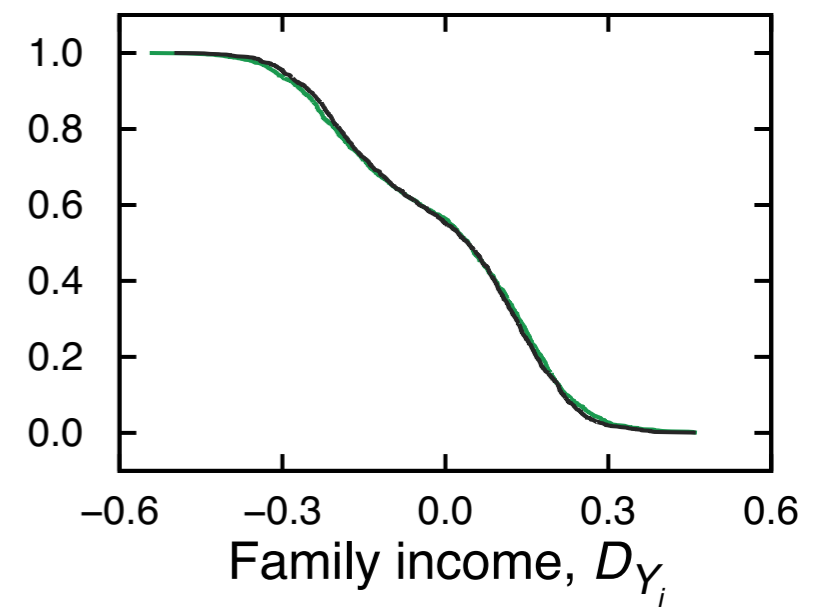

CDF

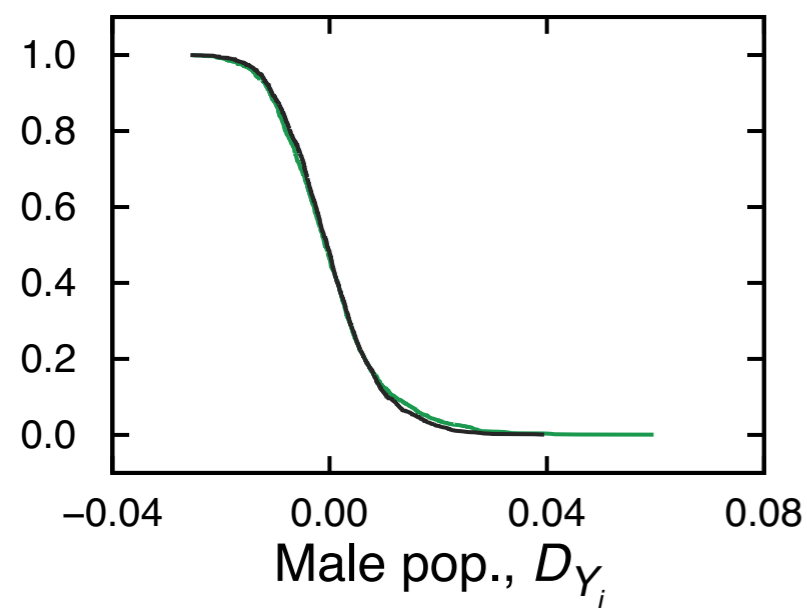

CDF

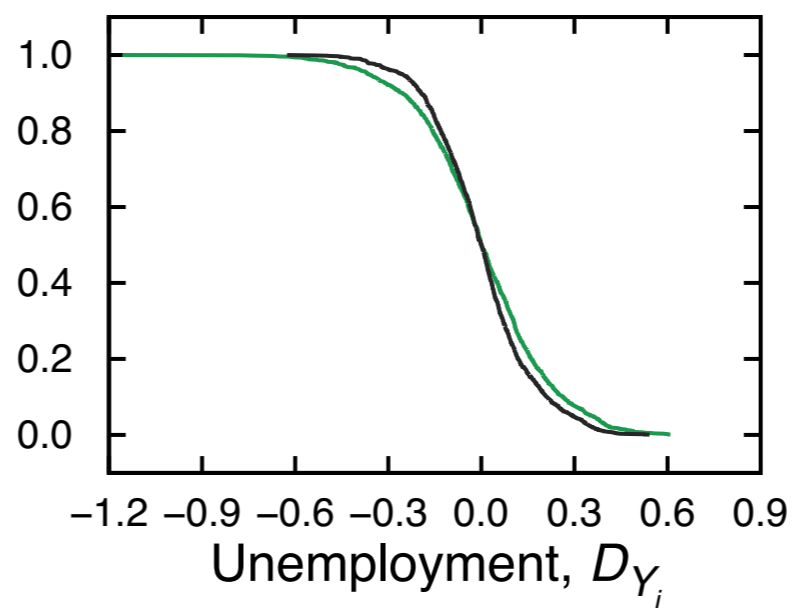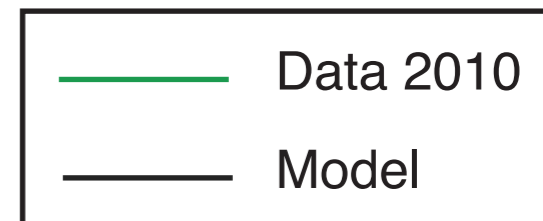

Supplement: S9 Fig — The same as S8 Fig considering data from the year of 2010. (PDF) [file pone.0134862.s010.pdf]
