# Supplementary material for: Scale-Adjusted Metrics for Predicting the Evolution of Urban Indicators and Quantifying the Performance of Cities
Source: PLoS One. 2015 Sep 10;10(9):e0134862. doi: 10.1371/journal.pone.0134862 (PMC4565645; doi:10.1371/journal.pone.0134862)
Supplement: S3 Fig — The purple dots show the values of D Yi(2000) versus D Yi(1991) for each city. The dashed lines are fits of the linear model D Yi(2000) = A i + α i D Yi(1991) (Eq 3) obtained via ordinary least-square regression. The values of α i and their standard errors are shown in the plots and also summarized in Table 2. (PDF) [file pone.0134862.s004.pdf]

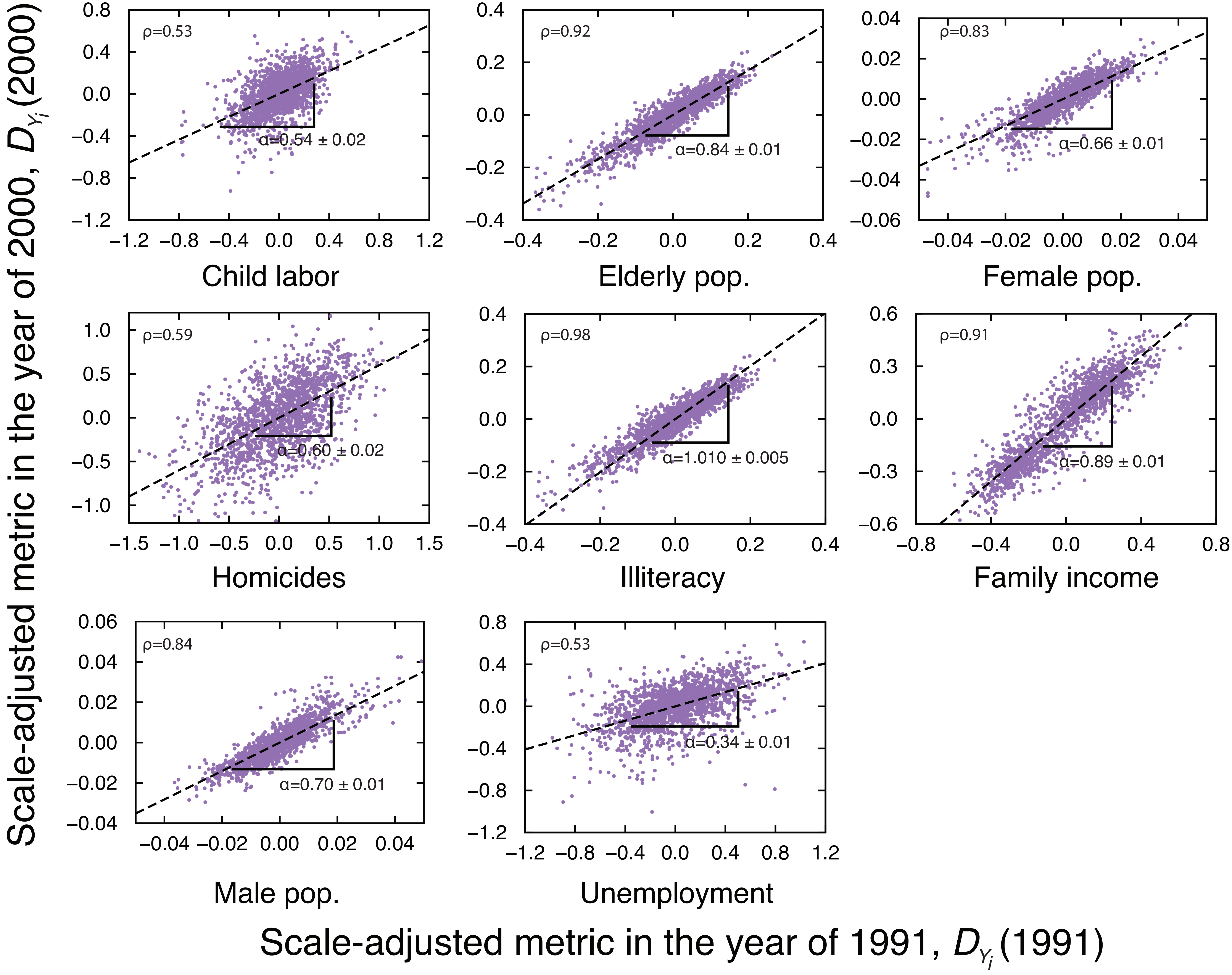

Scale-adjusted metric in the year of 2000, $D_{Y_i}(2000)$
ρ=0.53
α=0.54 ± 0.02
Child labor
ρ=0.92
α=0.84 ± 0.01
Elderly pop.
ρ=0.83
α=0.66 ± 0.01
Female pop.
ρ=0.59
α=0.60 ± 0.02
Homicides
ρ=0.98
α=1.010 ± 0.005
Illiteracy
ρ=0.91
α=0.89 ± 0.01
Family income
ρ=0.84
α=0.70 ± 0.01
Male pop.
ρ=0.53
α=0.34 ± 0.01
Unemployment
Scale-adjusted metric in the year of 1991, $D_{Y_i}(1991)$
