# Supplementary material for: Scale-Adjusted Metrics for Predicting the Evolution of Urban Indicators and Quantifying the Performance of Cities
Source: PLoS One. 2015 Sep 10;10(9):e0134862. doi: 10.1371/journal.pone.0134862 (PMC4565645; doi:10.1371/journal.pone.0134862)
Supplement: S6 Fig — These plots show the standard deviation σ of the scale-adjusted metrics D Yi(t + Δt) versus the average value of D Yi(t) evaluated in five equally spaced windows taken from the relationship between D Yi(t + Δt) and D Yi(t) (Fig 4 and S3 Fig) for the years 2000-1991 (left panel) and 2010-2000 (right panel). We note that the standard deviation can be approximated by a constant for most indicators in both combinations of years. We further observe that the small fluctuations in σ are probably the reason of why the Cramér von Mises test has rejected the normality of the fluctuations ξ shown in S5 Fig. When fitting the linear models of Eq 6, we have also taken into account this small heteroskedasticity (as implemented in the Stata 13—http://www.stata.com—via the robust option in the regress function) but the linear coefficients remain practically the same. (PDF) [file pone.0134862.s007.pdf]

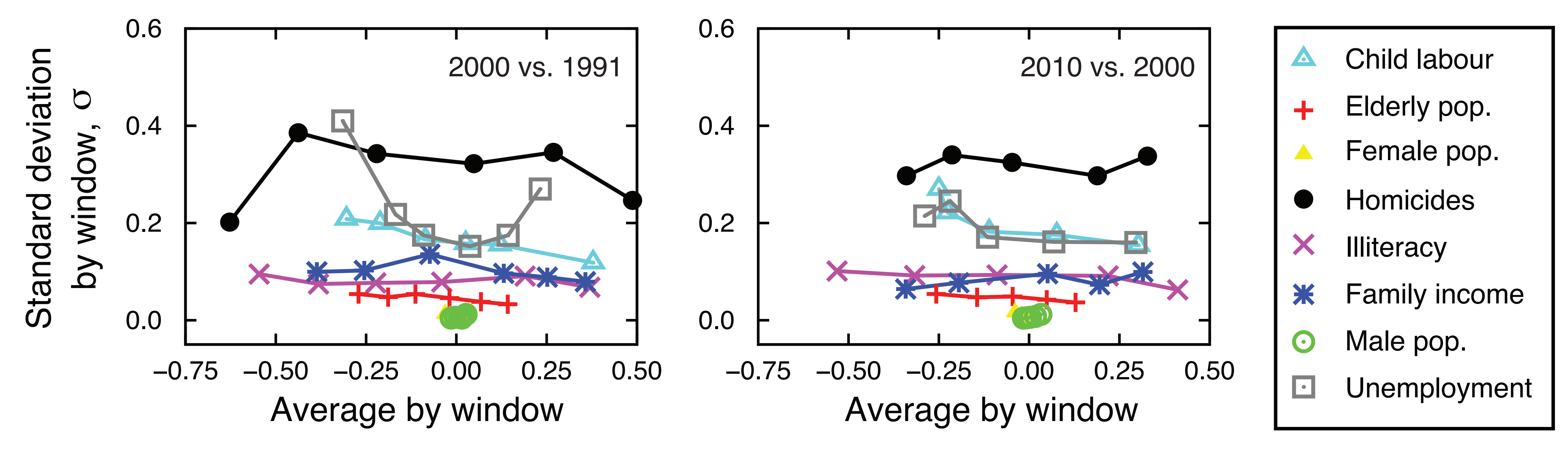

Standard deviation by window, σ
2000 vs. 1991
2010 vs. 2000
Average by window
0.6
0.4
0.2
0.0
–0.75
–0.50
–0.25
0.00
0.25
0.50
Child labour
Elderly pop.
Female pop.
Homicides
Illiteracy
Family income
Male pop.
Unemployment
