# Supplementary material for: Scale-Adjusted Metrics for Predicting the Evolution of Urban Indicators and Quantifying the Performance of Cities
Source: PLoS One. 2015 Sep 10;10(9):e0134862. doi: 10.1371/journal.pone.0134862 (PMC4565645; doi:10.1371/journal.pone.0134862)
Supplement: S7 Fig — We have applied the linear model of Eq 6 for predicting the values of D Yi(t) in the year of 2000 only using data from the year of 1991 as well as for predicting D Yi(t) in the year of 2010 only using data from the year of 2000. In both cases, we have calculated the average D Yi(t) for the predictions (gray bars) after grouping the cities in above (A) and below (B) the allometric laws (in that year) and compared these results with the same averages evaluated using the empirical data (blue bars for year of 2000 and green bars for the year of 2010). The errors bars are 95% bootstrapping confidence intervals for the average values. We observe that the predicted average values are in very good agreement with the empirical values for all urban indicators in both years. (PDF) [file pone.0134862.s008.pdf]

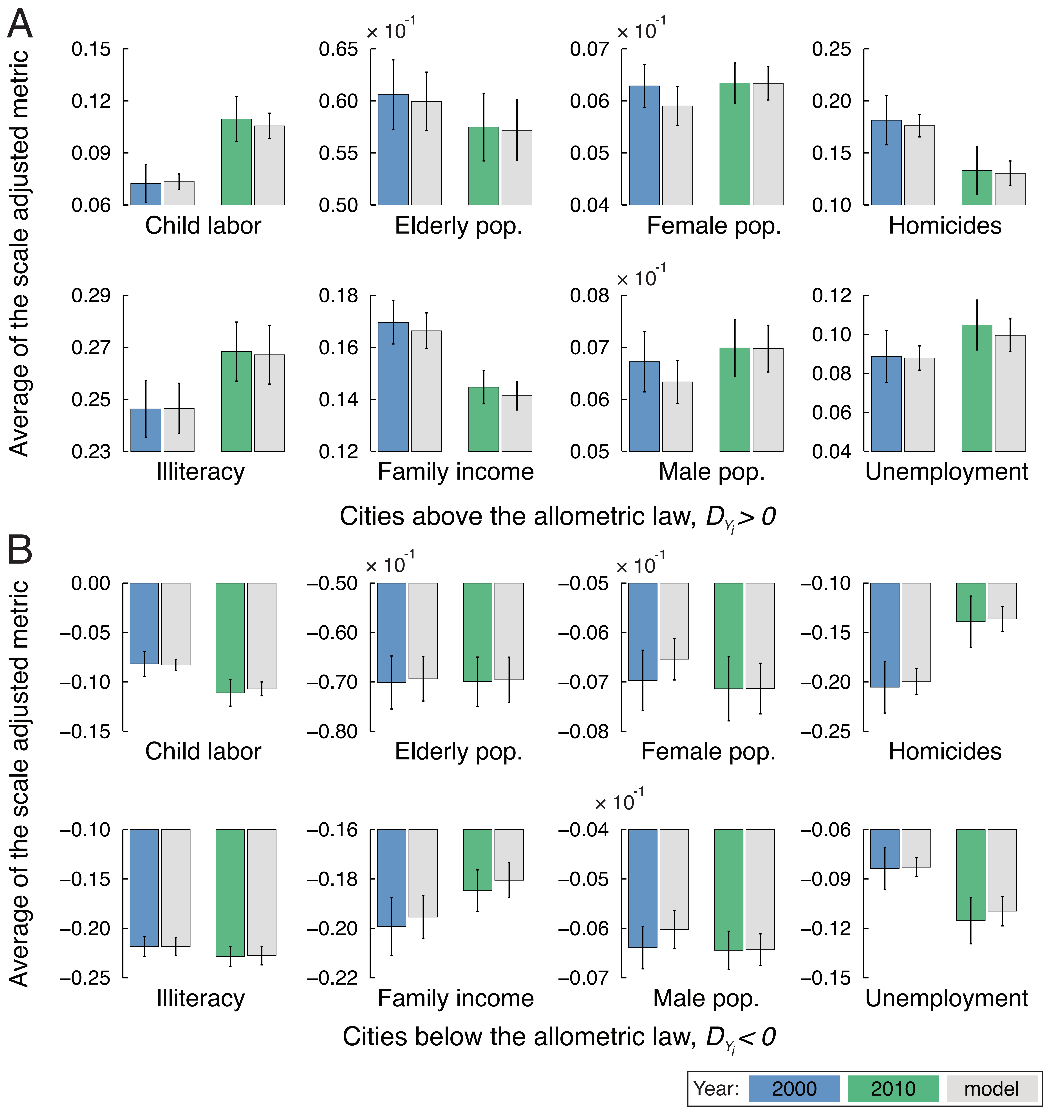

A
Average of the scale adjusted metric
Child labor
Elderly pop.
Female pop.
Homicides
Illiteracy
Family income
Male pop.
Unemployment
× 10^-1
Cities above the allometric law, $D_{Y_i} > 0$
B
Average of the scale adjusted metric
Child labor
Elderly pop.
Female pop.
Homicides
Illiteracy
Family income
Male pop.
Unemployment
× 10^-1
Cities below the allometric law, $D_{Y_i} < 0$
Year: 2000 2010 model
