# Supplementary material for: Scale-Adjusted Metrics for Predicting the Evolution of Urban Indicators and Quantifying the Performance of Cities
Source: PLoS One. 2015 Sep 10;10(9):e0134862. doi: 10.1371/journal.pone.0134862 (PMC4565645; doi:10.1371/journal.pone.0134862)
Supplement: S1 Text — Values of the linear coefficients in the model of the Eq 6 for the relationships D Yi(2000) versus D Yi(1991) and D Yi(2010) versus D Yi(2000) for the eight urban indicators. (PDF) [file pone.0134862.s011.pdf]

TABLE S1. **Regression model coefficients [$D_{Y_i}(2000)$ versus $D_{Y_i}(1991)$] for the indicator child labor.** Values of the linear coefficients C_k obtained via ordinary least-squares fit and standard errors. Here, z is the value of the $z-$statistic and p is the two-tail $p-$value for testing the hypothesis that the coefficient C_k is different from zero.

Indicator	Coefficient C_k	Standard Error	z-Statistic	$p > \|z\|$
Intercept, C_0	$< 10^{-4}$	0.0039	-0.0006	0.9995
Child labor	0.4968	0.0255	19.4809	$< 10^{-4}$
Elderly population	-0.1214	0.0518	-2.3424	0.0192
Female population	0.4145	3.6436	0.1138	0.9094
Homicides	-0.0158	0.0107	-1.4788	0.1392
Illiteracy	0.105	0.0257	4.0932	$< 10^{-4}$
Family income	-0.0031	0.0288	-0.1057	0.9158
Male population	-0.1512	3.8741	-0.039	0.9689
Unemployment	-0.0788	0.0152	-5.1896	$< 10^{-4}$
				Adjusted $R^2 = 0.3108$

TABLE S2. **Regression model coefficients [$D_{Y_i}(2000)$ versus $D_{Y_i}(1991)$] for the indicator elderly population.** Values of the linear coefficients C_k obtained via ordinary least-squares fit and standard errors. Here, z is the value of the $z-$statistic and p is the two-tail $p-$value for testing the hypothesis that the coefficient C_k is different from zero.

Indicator	Coefficient C_k	Standard Error	z-Statistic	$p > \|z\|$
Intercept, C_0	$< 10^{-4}$	0.0008	0.0035	0.9972
Child labor	0.0126	0.0054	2.3246	0.0201
Elderly population	0.8552	0.011	77.8926	$< 10^{-4}$
Female population	-2.2847	0.7718	-2.9601	0.0031
Homicides	-0.0077	0.0023	-3.398	0.0007
Illiteracy	-0.0203	0.0054	-3.7279	0.0002
Family income	-0.0188	0.0061	-3.0844	0.002
Male population	-2.1936	0.8207	-2.673	0.0075
Unemployment	-0.0225	0.0032	-7.0142	$< 10^{-4}$
				Adjusted $R^2 = 0.8634$

SUPPORTING INFORMATION

TABLE S3. **Regression model coefficients [$D_{Y_i}(2000)$ versus $D_{Y_i}(1991)$] for the indicator female population.** Values of the linear coefficients C_k obtained via ordinary least-squares fit and standard errors. Here, z is the value of the z−statistic and p is the two-tail p−value for testing the hypothesis that the coefficient C_k is different from zero.

Indicator	Coefficient C_k	Standard Error	z-Statistic	$p > \lvert z \rvert$
Intercept, C_0	$< 10^{-4}$	0.0001	0.0142	0.9887
Child labor	0.0034	0.0008	4.2271	$< 10^{-4}$
Elderly population	0.006	0.0016	3.6571	0.0003
Female population	-1.4729	0.1156	-12.7425	$< 10^{-4}$
Homicides	0.0013	0.0003	3.9537	0.0001
Illiteracy	-0.0033	0.0008	-4.0217	0.0001
Family income	0.0003	0.0009	0.2814	0.7784
Male population	-2.2567	0.1229	-18.3619	$< 10^{-4}$
Unemployment	0.0006	0.0005	1.1754	0.2398
			Adjusted $R^2 = 0.7444$	

TABLE S4. **Regression model coefficients [$D_{Y_i}(2000)$ versus $D_{Y_i}(1991)$] for the indicator homicides.** Values of the linear coefficients C_k obtained via ordinary least-squares fit and standard errors. Here, z is the value of the z−statistic and p is the two-tail p−value for testing the hypothesis that the coefficient C_k is different from zero.

Indicator	Coefficient C_k	Standard Error	z-Statistic	$p > \lvert z \rvert$
Intercept, C_0	$< 10^{-4}$	0.0078	-0.0001	0.9999
Child labor	0.1214	0.0515	2.3579	0.0184
Elderly population	-0.1557	0.1047	-1.4872	0.1369
Female population	-14.4108	7.3579	-1.9585	0.0502
Homicides	0.5291	0.0216	24.5214	$< 10^{-4}$
Illiteracy	0.3583	0.0518	6.914	$< 10^{-4}$
Family income	0.3293	0.0583	5.6529	$< 10^{-4}$
Male population	-14.8131	7.8233	-1.8935	0.0583
Unemployment	0.1086	0.0306	3.544	0.0004
			Adjusted $R^2 = 0.3859$	

TABLE S5. **Regression model coefficients [$D_{Y_i}(2000)$ versus $D_{Y_i}(1991)$] for the indicator illiteracy.** Values of the linear coefficients C_k obtained via ordinary least-squares fit and standard errors. Here, z is the value of the z−statistic and p is the two-tail p−value for testing the hypothesis that the coefficient C_k is different from zero.

Indicator	Coefficient C_k	Standard Error	z-Statistic	$p > \|z\|$
Intercept, C_0	$< 10^{-4}$	0.0013	-0.0013	0.999
Child labor	0.0172	0.0089	1.9389	0.0525
Elderly population	-0.0204	0.018	-1.1306	0.2582
Female population	-6.2844	1.2653	-4.9665	$< 10^{-4}$
Homicides	0.0035	0.0037	0.95	0.3421
Illiteracy	0.9723	0.0089	109.101	$< 10^{-4}$
Family income	-0.0518	0.01	-5.1722	$< 10^{-4}$
Male population	-7.6458	1.3454	-5.6831	$< 10^{-4}$
Unemployment	-0.0146	0.0053	-2.7745	0.0055
				Adjusted R^2 = 0.9617

TABLE S6. **Regression model coefficients [$D_{Y_i}(2000)$ versus $D_{Y_i}(1991)$] for the indicator family income.** Values of the linear coefficients C_k obtained via ordinary least-squares fit and standard errors. Here, z is the value of the z−statistic and p is the two-tail p−value for testing the hypothesis that the coefficient C_k is different from zero.

Indicator	Coefficient C_k	Standard Error	z-Statistic	$p > \|z\|$
Intercept, C_0	$< 10^{-4}$	0.0022	0.0007	0.9995
Child labor	0.103	0.0143	7.2094	$< 10^{-4}$
Elderly population	0.2216	0.029	7.6286	$< 10^{-4}$
Female population	6.7044	2.0421	3.2831	0.001
Homicides	0.0205	0.006	3.4157	0.0006
Illiteracy	-0.2024	0.0144	-14.075	$< 10^{-4}$
Family income	0.6953	0.0162	43.0002	$< 10^{-4}$
Male population	8.4487	2.1712	3.8912	0.0001
Unemployment	0.0076	0.0085	0.8895	0.3737
				Adjusted R^2 = 0.855

TABLE S7. **Regression model coefficients [$D_{Y_i}(2000)$ versus $D_{Y_i}(1991)$] for the indicator male population.** Values of the linear coefficients C_k obtained via ordinary least-squares fit and standard errors. Here, z is the value of the z–statistic and p is the two-tail p–value for testing the hypothesis that the coefficient C_k is different from zero.

Indicator	Coefficient C_k	Standard Error	z-Statistic	$p > \|z\|$
Intercept, C_0	$< 10^{-4}$	0.0001	-0.0072	0.9942
Child labor	-0.0029	0.0008	-3.8079	0.0001
Elderly population	-0.0056	0.0016	-3.5526	0.0004
Female population	1.6953	0.11	15.4191	$< 10^{-4}$
Homicides	-0.0013	0.0003	-4.1151	$< 10^{-4}$
Illiteracy	0.0035	0.0008	4.4749	$< 10^{-4}$
Family income	-0.0001	0.0009	-0.1618	0.8714
Male population	2.4809	0.1169	21.2214	$< 10^{-4}$
Unemployment	-0.0004	0.0005	-0.7999	0.4238
				Adjusted $R^2 = 0.7584$

TABLE S8. **Regression model coefficients [$D_{Y_i}(2000)$ versus $D_{Y_i}(1991)$] for the indicator unemployment.** Values of the linear coefficients C_k obtained via ordinary least-squares fit and standard errors. Here, z is the value of the z–statistic and p is the two-tail p–value for testing the hypothesis that the coefficient C_k is different from zero.

Indicator	Coefficient C_k	Standard Error	z-Statistic	$p > \|z\|$
Intercept, C_0	$< 10^{-4}$	0.004	-0.0014	0.9989
Child labor	-0.2975	0.0261	-11.3884	$< 10^{-4}$
Elderly population	-0.0839	0.0531	-1.5809	0.1139
Female population	18.4065	3.7325	4.9314	$< 10^{-4}$
Homicides	0.0294	0.0109	2.6875	0.0072
Illiteracy	0.2131	0.0263	8.1051	$< 10^{-4}$
Family income	0.2568	0.0296	8.6892	$< 10^{-4}$
Male population	17.6686	3.9686	4.4521	$< 10^{-4}$
Unemployment	0.2305	0.0155	14.8298	$< 10^{-4}$
				Adjusted $R^2 = 0.3641$

TABLE S9. **Regression model coefficients** [$D_{Y_i}(2010)$ **versus** $D_{Y_i}(2000)$] **for the indicator child labor.** Values of the linear coefficients C_k obtained via ordinary least-squares fit and standard errors. Here, z is the value of the z−statistic and p is the two-tail p−value for testing the hypothesis that the coefficient C_k is different from zero.

Indicator	Coefficient C_k	Standard Error	z-Statistic	$p > \|z\|$
Intercept, C_0	$< 10^{-4}$	0.0041	-0.004	0.9968
Child labor	0.5655	0.0264	21.3901	$< 10^{-4}$
Elderly population	-0.079	0.0565	-1.3984	0.162
Female population	21.5857	8.2891	2.6041	0.0092
Homicides	0.0102	0.0112	0.915	0.3602
Illiteracy	0.1443	0.0282	5.1266	$< 10^{-4}$
Family income	0.0381	0.0327	1.1655	0.2438
Male population	23.4009	8.4723	2.7621	0.0057
Unemployment	-0.2632	0.0246	-10.7168	$< 10^{-4}$
				Adjusted $R^2 = 0.4464$

TABLE S10. **Regression model coefficients** [$D_{Y_i}(2010)$ **versus** $D_{Y_i}(2000)$] **for the indicator elderly population.** Values of the linear coefficients C_k obtained via ordinary least-squares fit and standard errors. Here, z is the value of the z−statistic and p is the two-tail p−value for testing the hypothesis that the coefficient C_k is different from zero.

Indicator	Coefficient C_k	Standard Error	z-Statistic	$p > \|z\|$
Intercept, C_0	$< 10^{-4}$	0.0007	0.0041	0.9967
Child labor	0.0284	0.0047	6.0594	$< 10^{-4}$
Elderly population	0.9171	0.01	91.6951	$< 10^{-4}$
Female population	-2.2613	1.4681	-1.5403	0.1235
Homicides	0.0025	0.002	1.2593	0.2079
Illiteracy	-0.0679	0.005	-13.6101	$< 10^{-4}$
Family income	-0.0003	0.0058	-0.0574	0.9542
Male population	-2.1019	1.5005	-1.4008	0.1613
Unemployment	0.0052	0.0043	1.1985	0.2307
				Adjusted $R^2 = 0.889$

TABLE S11. **Regression model coefficients [$D_{Y_i}(2010)$ versus $D_{Y_i}(2000)$] for the indicator female population.** Values of the linear coefficients C_k obtained via ordinary least-squares fit and standard errors. Here, z is the value of the z−statistic and p is the two-tail p−value for testing the hypothesis that the coefficient C_k is different from zero.

Indicator	Coefficient C_k	Standard Error	z-Statistic	$p > \|z\|$
Intercept, C_0	$< 10^{-4}$	0.0001	-0.0105	0.9916
Child labor	-0.0003	0.0007	-0.4534	0.6503
Elderly population	0.0066	0.0016	4.1671	$< 10^{-4}$
Female population	0.8935	0.2339	3.8196	0.0001
Homicides	0.0001	0.0003	0.4459	0.6557
Illiteracy	-0.0021	0.0008	-2.598	0.0094
Family income	-0.0016	0.0009	-1.7511	0.0799
Male population	0.0215	0.2391	0.09	0.9283
Unemployment	0.0004	0.0007	0.5594	0.5759
				Adjusted R^2 = 0.7865

TABLE S12. **Regression model coefficients [$D_{Y_i}(2010)$ versus $D_{Y_i}(2000)$] for the indicator homicides.** Values of the linear coefficients C_k obtained via ordinary least-squares fit and standard errors. Here, z is the value of the z−statistic and p is the two-tail p−value for testing the hypothesis that the coefficient C_k is different from zero.

Indicator	Coefficient C_k	Standard Error	z-Statistic	$p > \|z\|$
Intercept, C_0	$< 10^{-4}$	0.0069	-0.0017	0.9987
Child labor	0.0682	0.0449	1.5195	0.1286
Elderly population	-0.862	0.0958	-8.994	$< 10^{-4}$
Female population	6.5982	14.0685	0.469	0.6391
Homicides	0.3028	0.0189	15.9874	$< 10^{-4}$
Illiteracy	0.5673	0.0478	11.8719	$< 10^{-4}$
Family income	0.1361	0.0556	2.4497	0.0143
Male population	3.337	14.3795	0.2321	0.8165
Unemployment	0.189	0.0417	4.5345	$< 10^{-4}$
				Adjusted R^2 = 0.395

TABLE S13. **Regression model coefficients [$D_{Y_i}(2010)$ versus $D_{Y_i}(2000)$] for the indicator illiteracy.** Values of the linear coefficients C_k obtained via ordinary least-squares fit and standard errors. Here, z is the value of the z−statistic and p is the two-tail p−value for testing the hypothesis that the coefficient C_k is different from zero.

Indicator	Coefficient C_k	Standard Error	z-Statistic	$p > \|z\|$
Intercept, C_0	$< 10^{-4}$	0.0013	0.0008	0.9994
Child labor	0.0224	0.0087	2.5747	0.01
Elderly population	0.0466	0.0186	2.5032	0.0123
Female population	0.7564	2.7333	0.2767	0.782
Homicides	0.0194	0.0037	5.2725	$< 10^{-4}$
Illiteracy	0.9848	0.0093	106.076	$< 10^{-4}$
Family income	-0.0815	0.0108	-7.551	$< 10^{-4}$
Male population	0.8701	2.7937	0.3115	0.7554
Unemployment	-0.0112	0.0081	-1.3776	0.1683
				Adjusted R^2 = 0.9664

TABLE S14. **Regression model coefficients [$D_{Y_i}(2010)$ versus $D_{Y_i}(2000)$] for the indicator family income.** Values of the linear coefficients C_k obtained via ordinary least-squares fit and standard errors. Here, z is the value of the z−statistic and p is the two-tail p−value for testing the hypothesis that the coefficient C_k is different from zero.

Indicator	Coefficient C_k	Standard Error	z-Statistic	$p > \|z\|$
Intercept, C_0	$< 10^{-4}$	0.0015	-0.0017	0.9986
Child labor	0.0411	0.0095	4.3127	$< 10^{-4}$
Elderly population	0.1079	0.0204	5.2987	$< 10^{-4}$
Female population	2.3141	2.9885	0.7743	0.4387
Homicides	0.0014	0.004	0.3363	0.7366
Illiteracy	-0.1401	0.0102	-13.8011	$< 10^{-4}$
Family income	0.6639	0.0118	56.2639	$< 10^{-4}$
Male population	1.4591	3.0545	0.4777	0.6329
Unemployment	0.0194	0.0089	2.1912	0.0284
				Adjusted R^2 = 0.9073

TABLE S15. **Regression model coefficients [$D_{Y_i}(2010)$ versus $D_{Y_i}(2000)$] for the indicator male population.** Values of the linear coefficients C_k obtained via ordinary least-squares fit and standard errors. Here, z is the value of the z–statistic and p is the two-tail p–value for testing the hypothesis that the coefficient C_k is different from zero.

Indicator	Coefficient C_k	Standard Error	z-Statistic	$p > \|z\|$
Intercept, C_0	$< 10^{-4}$	0.0001	0.0192	0.9847
Child labor	0.0005	0.0007	0.6436	0.5198
Elderly population	-0.0068	0.0015	-4.4126	$< 10^{-4}$
Female population	0.0079	0.2246	0.0352	0.9719
Homicides	-0.0002	0.0003	-0.5066	0.6125
Illiteracy	0.0022	0.0008	2.9033	0.0037
Family income	0.0017	0.0009	1.8973	0.0578
Male population	0.887	0.2295	3.8645	0.0001
Unemployment	-0.0005	0.0007	-0.7457	0.4559
				Adjusted R^2 = 0.7958

TABLE S16. **Regression model coefficients [$D_{Y_i}(2010)$ versus $D_{Y_i}(2000)$] for the indicator unemployment.** Values of the linear coefficients C_k obtained via ordinary least-squares fit and standard errors. Here, z is the value of the z–statistic and p is the two-tail p–value for testing the hypothesis that the coefficient C_k is different from zero.

Indicator	Coefficient C_k	Standard Error	z-Statistic	$p > \|z\|$
Intercept, C_0	$< 10^{-4}$	0.0033	0.0015	0.9988
Child labor	-0.2626	0.0217	-12.1156	$< 10^{-4}$
Elderly population	-0.1242	0.0463	-2.6826	0.0073
Female population	-3.6092	6.7955	-0.5311	0.5953
Homicides	0.0013	0.0091	0.1461	0.8838
Illiteracy	0.3983	0.0231	17.2582	$< 10^{-4}$
Family income	0.0978	0.0268	3.6456	0.0003
Male population	-3.9956	6.9457	-0.5753	0.5651
Unemployment	0.5028	0.0201	24.9752	$< 10^{-4}$
				Adjusted R^2 = 0.601
